# Regulation of GLI1 by cis DNA elements and epigenetic marks

**DOI:** 10.1016/j.dnarep.2019.04.011

**Published:** 2019-05-02

**Authors:** Robert Taylor, Jun Long, Joon Won Yoon, Ronnie Childs, Kathrine B. Sylvestersen, Michael L. Nielsen, King-Fu Leong, Stephen Iannaccone, David O. Walterhouse, David J. Robbins, Philip Iannaccone

**Affiliations:** a Developmental Biology Program, Stanley Manne Children’s Research Institute, Department of Pediatrics, Ann & Robert H. Lurie Children’s Hospital, Northwestern University Feinberg School of Medicine, USA; b The DeWitt Daughtry Family Department of Surgery, Miller School of Medicine, University of Miami, USA; c Center for Protein Research, University of Copenhagen, DK

**Keywords:** Hedgehog signaling pathway, Oncogene, Transcription factor, Epigenetic, DNA-protein interaction

## Abstract

GLI1 is one of three transcription factors (GLI1, GLI2 and GLI3) that mediate the Hedgehog signal transduction pathway and play important roles in normal development. GLI1 and GLI2 form a positive-feedback loop and function as human oncogenes. The mouse and human GLI1 genes have untranslated 5′ exons and large introns 5′ of the translational start. Here we show that Sonic Hedgehog (SHH) stimulates occupancy in the introns by H3K27ac, H3K4me3 and the histone reader protein BRD4. H3K27ac and H3K4me3 occupancy is not significantly changed by removing BRD4 from the human intron and transcription start site (TSS) region. We identified six GLI binding sites (GBS) in the first intron of the human GLI1 gene that are in regions of high sequence conservation among mammals. GLI1 and GLI2 bind all of the GBS *in vitro*. Elimination of GBS1 and 4 attenuates transcriptional activation by GLI1. Elimination of GBS1, 2, and 4 attenuates transcriptional activation by GLI2. Eliminating all sites essentially eliminates reporter gene activation. Further, GLI1 binds the histone variant H2A.Z. These results suggest that GLI1 and GLI2 can regulate GLI1 expression through protein-protein interactions involving complexes of transcription factors, histone variants, and reader proteins in the regulatory intron of the GLI1 gene. GLI1 acting in trans on the GLI1 intron provides a mechanism for GLI1 positive feedback and auto-regulation. Understanding the combinatorial protein landscape in this locus will be important to interrupting the GLI positive feedback loop and providing new therapeutic approaches to cancers associated with GLI1 overexpression.

## 1. Introduction

The Sonic Hedgehog/Patched/GLI pathway is important for normal development, cancer and congenital anomalies [1-3]. It is a complex signal transduction pathway involving reversal of negative regulation of a seven span transmembrane protein, Smoothened (SMO) by the receptor Patched (PTCH) following stimulation by the ligand Sonic Hedgehog (SHH). SHH signaling is mediated by three transcription factors, GLI1, GLI2 and GLI3. Without SHH ligand transcriptional activity of these factors is repressed by Suppressor of Fused (SuFu). SuFu binds the GLI factors preventing them from moving to the nucleus. SHH signaling activity is located in the primary cilium of the cell where the presence of SHH ligand causes PTCH to be endocytosed internalizing the SHH/PTCH complex and preventing PTCH from sequestering SMO intracellularly. PTCH then does not inhibit SMO, induces SMO to localize in the base of the primary cilium, and represses SuFu. SHH signaling, through PTCH/SMO, recruits GLI/SuFu to the ciliary tip where they are rapidly dissociated [4]. This promotes nuclear localization of the GLI transcription factors allowing target gene regulation [5-7]. SuFu may also act as a chaperon to the GLI transcription factors, participating in chromatin complexes and facilitating import and export of GLI factors from the nucleus [8].

A significant disease burden is associated with mis-regulation of this signal transduction pathway [1]. Constitutive signaling activity is associated with cancers or birth defects while decreased signaling activity is associated with birth defects. GLI1 is a human oncogene and constitutive activation, either as a result of canonical pathway activation or non-canonical activation, of GLI1 is associated with many human cancers [9-11]. Clinical trials have been conducted to establish the efficacy of down-regulating the pathway in patients by interfering with SMO [12,13]. Unfortunately this approach may not be effective in many patients because of non-canonical signaling, which directly activates GLI1 and bypasses the block at the level of SMO. Thus there is a need to target GLI1 to more effectively block the pathway and optimize the chance for therapeutic efficacy. In order to ameliorate the disease burden associated with this gene pathway and thereby improve the public health, we need to more fully understand how GLI1 regulates transcription and what regulates GLI1 expression.

GLI1 is transcriptionally activated by GLI2, and non-canonical signaling, independent of SHH, PTCH and SMO, has been observed through several pathways (KRAS, TGFbeta, WNT) and with transcription factors, including c-MYC and EWSR1-FLI1, that directly activate transcription of GLI1 [14-18]. Importantly GLI1 expression feeds forward inducing GLI1 expression [19]. Negative regulation of the feedback loop can be provided by GLI3, translational repression or by the repressive effects of the lncRNA, GLI1as (GLI1 antisense long noncoding RNA) [20].

The interplay of chromatin landscape and transcription factors is key to regulation of gene expression. It is well established that epigenetic modification of histones correlates with transcriptional activity. In particular H3K27ac and H3K4me3 are present at sites of active transcription [21]. Altered histones are believed to be “sensed” by a class of proteins known collectively as reader proteins [22,23]. These include bromodomain proteins that recognize acetylation marks on histones. Their role in transcriptional regulation remains to be fully elucidated but their ability to bind modified histones is sufficiently important to transcriptional regulation to motivate the development of small molecule inhibitors to modulate activity in cancers [24]. A particularly important bromodomain reader protein, BRD4, binds histone H3 and H4 acetylated tails as well as histone methylases [25]. BRD4 is an important regulator of gene activity in several cancers [26-28].

Given the widespread association of GLI1 with many human cancers including glioblastoma, basal cell carcinoma, lung cancer, GI cancers, prostate cancer [1], medulloblastoma [11,29,30] and rhabdomyosarcoma [31-33], it is important to understand key elements of the regulation of GLI1 expression. Here we show that the intronic region 5′ of the GLI1 translational start is a regulatory region with chromatin features of an enhancer. The first intron in the human locus contains numerous cis elements, and is associated with H3K27ac marks and DNase hypersensitivity clusters. We show that SHH stimulates occupancy in this region by H3K27ac, H3K4me3 and the reader protein BRD4 and that BRD4 can be removed from the human intron and TSS region without resulting in lower H3K27ac and H3K4me3 occupancy. Our sequencing a number of years ago and public reference sequence data show six conserved GLI binding sites (GBS) in the large first intron of the human GLI1 gene. We show that these six GBS in the first intron of the human GLI1 gene are in regions of high sequence conservation among mammals. Elimination of GBS1 and 4 attenuates transcriptional activation by GLI1. Elimination of GBS1, 2, and 4 attenuates transcriptional activation by GLI2. Deletions of combinations of the GBS indicate a hierarchy of importance of the sites. Eliminating all sites eliminates target gene activation. GLI1 and GLI2 bind the sites and GLI1 binds the histone variant H2A.Z. The results suggest that GLI1 expression can be auto-regulated by the GLI genes GLI1 and GLI2 through protein complex binding to this region of the GLI1 gene. Elucidating the combinatorial protein-protein interactions that regulate GLI1 expression will provide new therapeutic approaches to GLI1 induced cancers overcoming the shortfall of upstream inhibitors whose efficacy is limited by non-canonical signaling.

## 2. Results

### 2.1. The first intron of human GLI1 has the characteristics of an enhancer and contains conserved putative GLI binding sites (GBS)

**Fig. 1.**
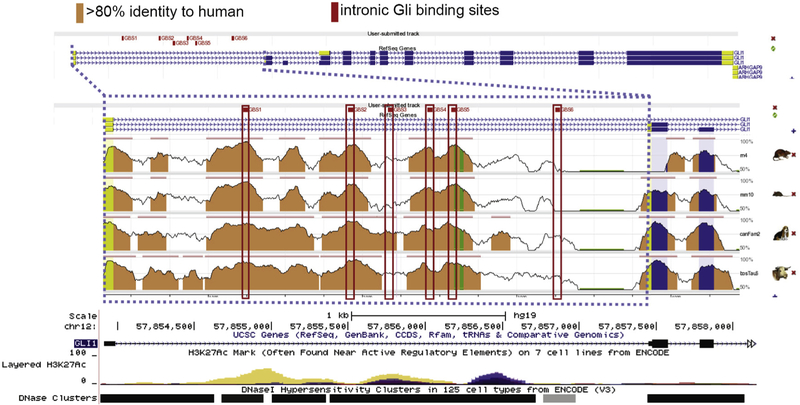
The human GLI1 first intron (outlined with blue dashed lines) has the genomic characteristics of an enhancer. Public data and our sequencing reveal six GLI binding sites (GBS) with 8/9 consensus nt in regions of very high sequence conservation between mammalian species. Red boxes indicate the GBS; conservation peaks with reference to the human sequence are shown; salmon indicates > 80% sequence identity in 100 nt windows, TSS is the 5′ end of the first exon and the translational start is the 5′ end of the second exon. Peaks of H3K27ac and DNase hypersensitivity clusters are shown. Additional analysis and transcription factor ChIP seq plots are provided in Supplemental materials Fig. 1.

The first exon of the human GLI1 gene is non-coding and the ATG translational start is at the 5′ end of the second exon [34]. The first intron of the human GLI1 gene is highly conserved among mammals (Fig. 1). The region has several DNase hypersensitivity clusters, indicating open chromatin, and H3K27ac marks in multiple cell lines. Transcription Factor ChIP-seq from ENCODE identified 161 factors in this region and Chromatin State Segmentation by HMM demonstrated the human intron is a regulatory region in nine cell lines. Histone modifications by ChIP-seq from ENCODE showed areas of high signal from CTCF, H3Kme1 and H3K27ac in human stem cells (H1) and in CML cells (K562) (Fig. 1, and Fig. 1 in Supplemental materials).

### 2.2. Epigenetic markers of gene activation and GLI2 binding are stimulated in the mouse GLI1 second intron (equivalent to human first intron) by SHH signaling

**Fig. 2.**
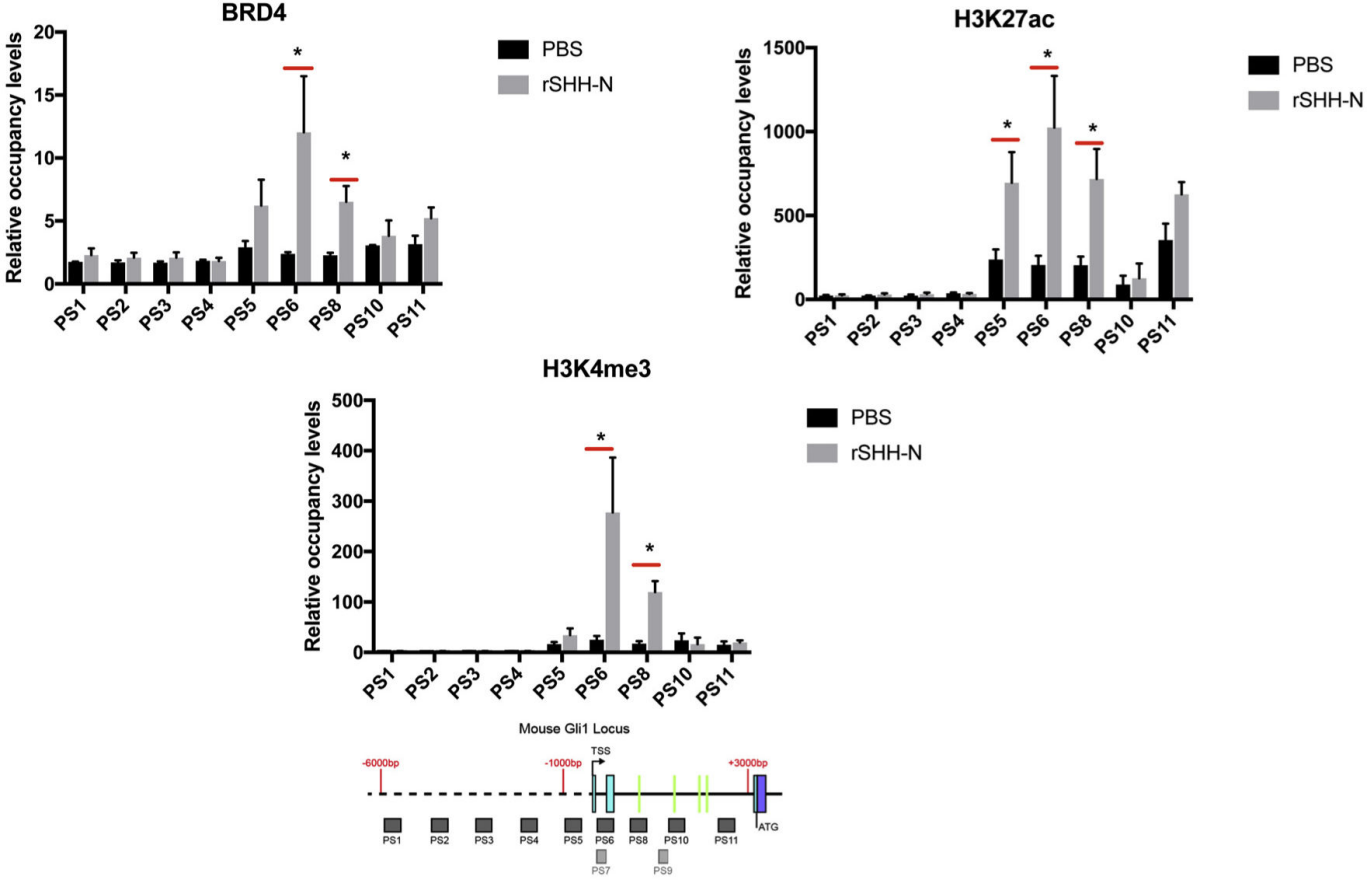
Epigenetic marks are present in the second intron of the mouse GLI1 locus upon SHH stimulation. BRD4, H3K27ac and H3K4me3 occupancy were analyzed by ChIP-quantitative PCR in SHH responsive cells (mouse LT2) treated with 1 μg/μl recombinant SHH-N (C25II Substitution) for 24 h. The signals were normalized to a control ChIP performed using rabbit IgG. Error bars represent the S.E. of three independent experiments. p values ≤ 0.05 are considered statistically significant and indicated by an asterisk. (Below) a schematic of the mouse GLI1 locus from −6,000 nt to + 3000 nt, relative to the transcription start sites (TSS) is shown. The probes PS1-PS11 are represented as shaded boxes (sequences in Methods section). 5′ - > 3′ is left to right. The solid line represents introns, the dashed line represents upstream sequence and the colored boxes represent exons. Green lines in the intron represent the locations of the mouse GBS.

In order to interrogate the occupancy of the region by activating epigenetic histone marks and their response to SHH we utilized mouse LIGHT2 (LT2) [36] cells because of their known, well-characterized response to SHH in the presence of intact SHH/GLI1 signaling. The mouse GLI1 sequence previously described by us includes an additional exon relative to the human sequence with a short first intron, making the mouse second intron equivalent to the human first intron [34]. SHH activates GLI1 expression [35]. Indications that the intronic region is an active regulatory region led us to look for evidence that SHH increases transcriptional activation through the proximal promoter and intronic region of GLI1 in human and mouse cells (Fig. 2).

**Fig. 3.**
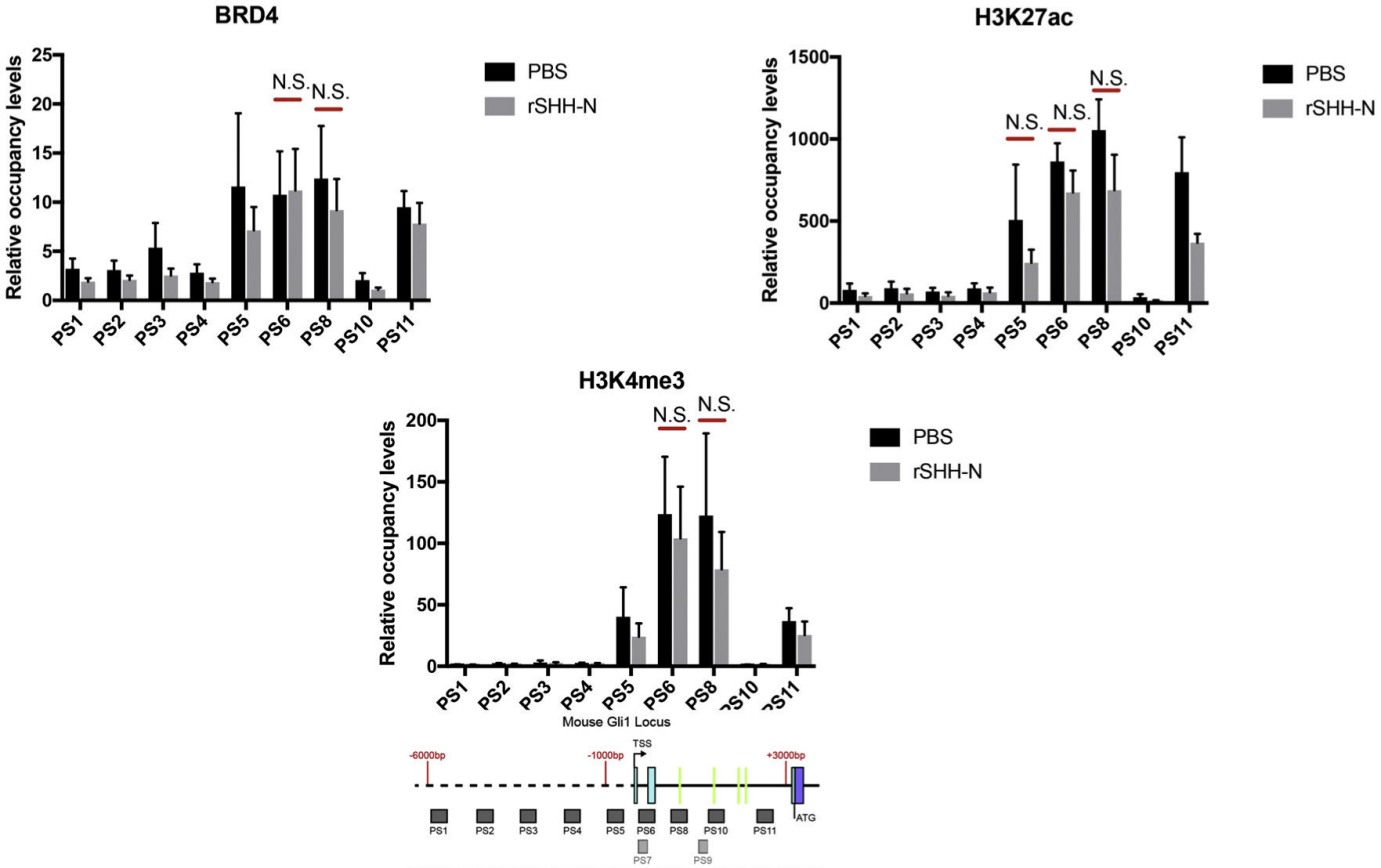
Epigenetic marks are not responsive to SHH stimulation in cells that lack GLI2 and GLI3 (Gli2−/−;Gli3−/− MEFs). Brd4, H3K27ac and H3K4me3 occupancy in the region were analyzed by ChIP-quantitative PCR in Gli2−/−;Gli3−/− MEFs treated with 1 μg/μl recombinant SHH-N (C25II Substitution) for 24 h. The signals were normalized to a control ChIP performed using rabbit IgG. Error bars represent the S.E. of three independent experiments. p values ≤ 0.05 are considered statistically significant and indicated by an asterisk. (Below) a schematic of the mouse GLI1 locus from −6,000 nt to + 3000 nt, relative to the transcription start sites (TSS) is shown. The probe sets (PS) PS1-PS11 are represented as shaded boxes (sequences in Methods section). 5′ - > 3′ is left to right. Green lines in the intron represent the locations of the mouse GBS.

LT2 [36] cells demonstrated stimulation of occupancy of the TSS region and introns by BRD4, H3K27ac and H3K4me3 with recombinant SHH. We then wished to determine if this SHH responsiveness was dependent on GLI and to accomplish this we used GLI2/3 (−/−) mouse embryonic fibroblasts (MEF). BRD4, H3K27ac and H3K4me3 occupied this region in the absence of GLI2 and GLI3, however, SHH stimulation of occupancy was not seen in cells lacking GLI2 and GLI3 (Fig. 3).

**Fig. 4.**
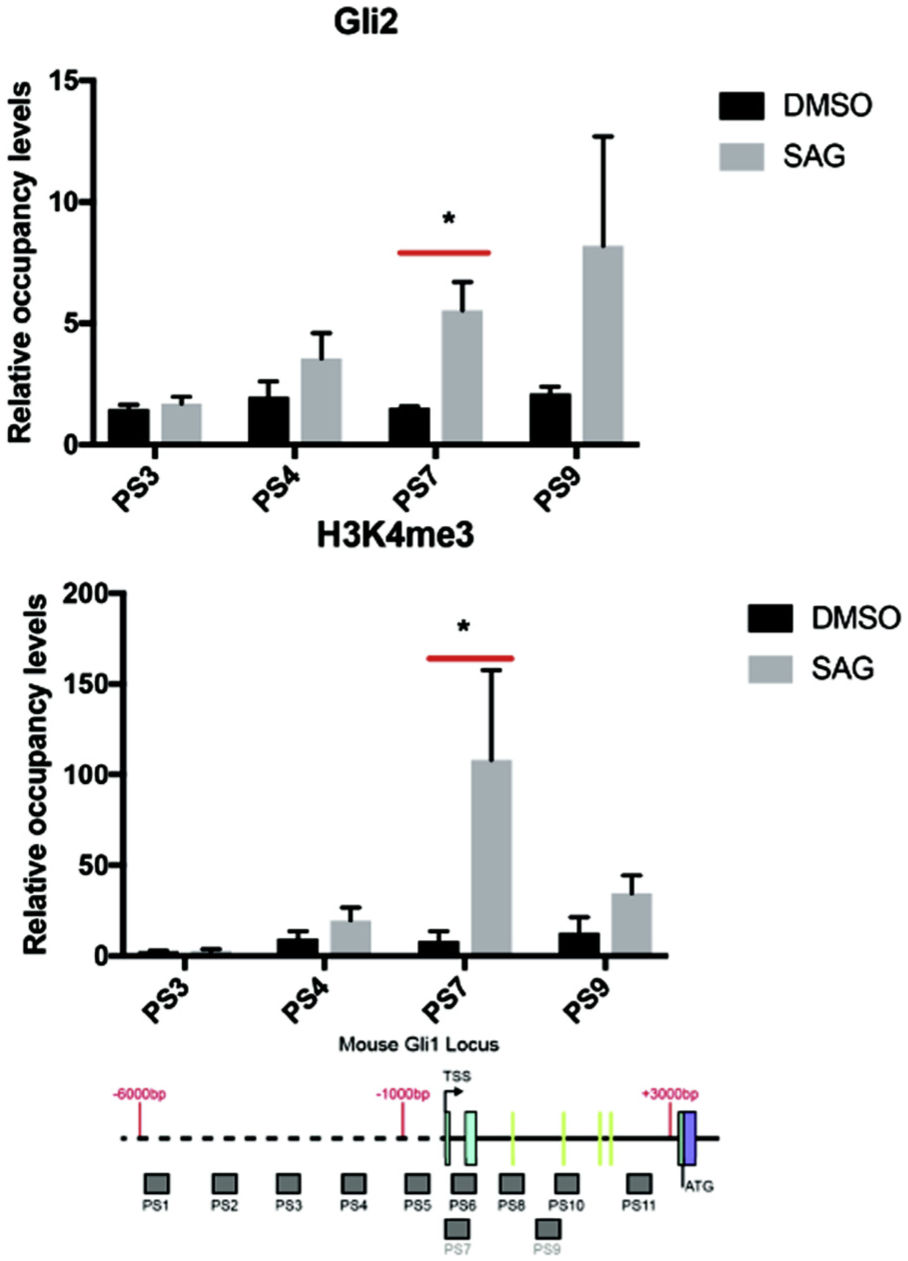
GLI2 and H3K4me3 are enriched in the GLI1 locus with SMO agonist (SAG) stimulation in LT2 SHH responsive mouse cells by ChIP-quantitative PCR. The signals were normalized to a control ChIP performed using rabbit IgG. Error bars represent the S.E. of three independent experiments. p values ≤ 0.05 are considered statistically significant and indicated by an asterisk. (Below) a schematic of the mouse GLI1 locus from −6,000 nt to + 3000 nt, relative to the transcription start sites (TSS) is shown. The probe sets (PS) PS1-PS11 are represented as shaded boxes (sequences in Methods section). 5′ - > 3′ is left to right. Green lines in the intron represent the locations of the mouse GBS.

The highest occupancy by BRD4, H3K27ac and H3K4me3 occur around the TSS and the 5′ end of the mouse first and second intron (Figs. 2, 3). In order to establish if the SHH responsiveness could be upstream of SMO we stimulated LT2 cells (that have intact SMO/GLI signaling) with SMO agonist. H3K4me3 occupancy in the TSS region and in the introns was increased with SMO agonist (SAG). Further, GLI2 occupancy was increased by SAG in the region of the TSS (Fig. 4).

**Fig. 5.**
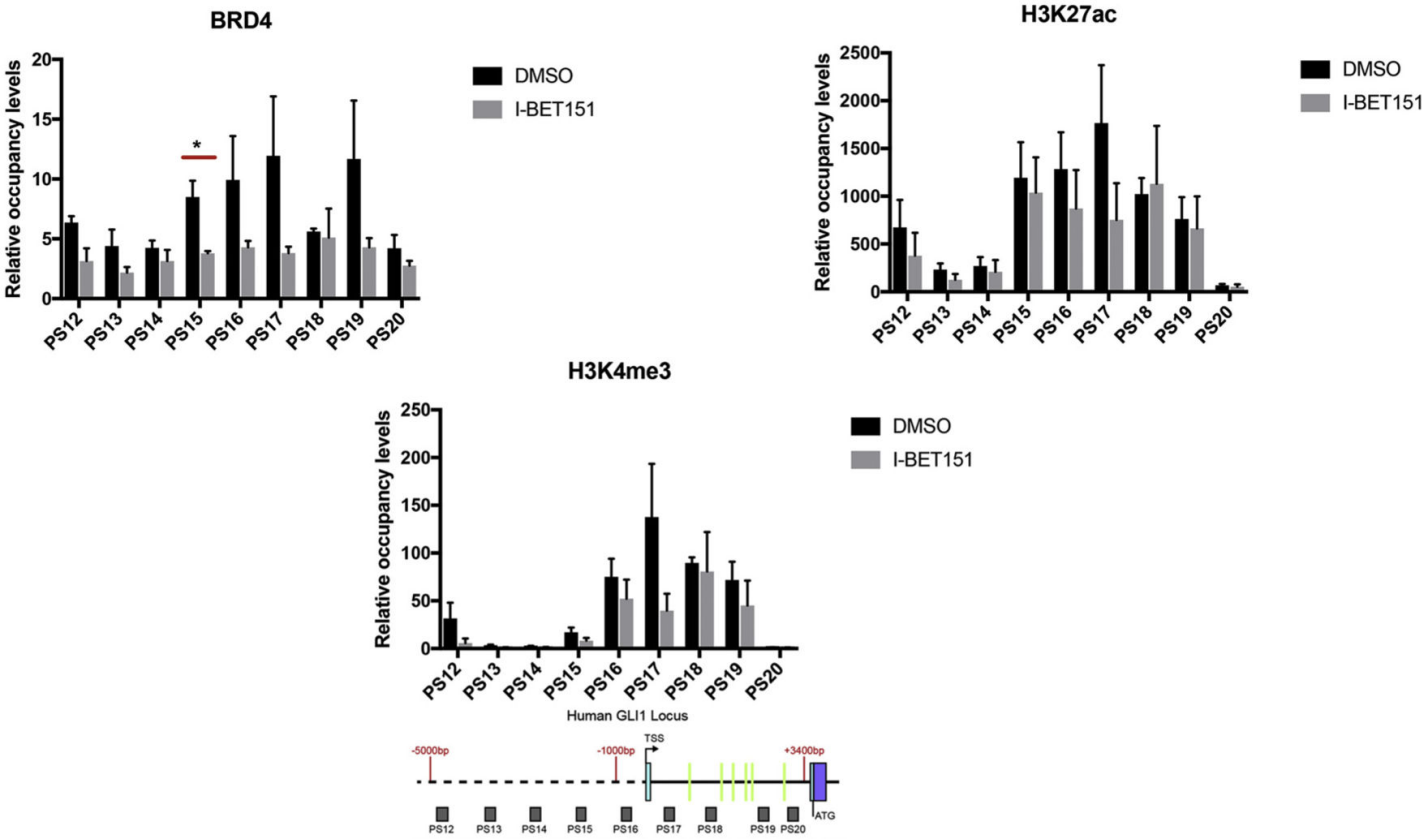
BRD4 occupancy is reduced with treatment by I-BET151 in the human GLI1 locus. Brd4, H3K27ac and H3K4me3 occupancy were analyzed by ChIP-quantitative PCR in BL1648 (human Burkitt lymphoma cells not dependent on SHH signaling) cells treated with 1μM I-BET151 for 24 h. The signals were normalized to a control ChIP performed using rabbit IgG. I-BET151 (an inhibitor of bromodomain end terminal protein) reduces BRD4 without changing active chromatin mark occupancy in the first intron of the human GLI1 locus. Error bars represent the S.E. of three independent experiments, unless otherwise indicated. p values ≤ 0.05 are considered statistically significant and indicated by an asterisk. (Below) A schematic of the human GLI1 locus from −5,000 nt to + 3700 nt, relative to the transcription start site (TSS) is shown. The probe sets (PS) PS12-PS20 are represented as shaded boxes (sequences in Methods section). 5′ - > 3′ is left to right. Green lines in the intron represent the locations of the human GBS.

Because inhibition of BRD4 binding by I-BET151 in mouse cells reduced GLI1 expression and is a candidate cancer therapy [28] we wished to determine the effect of I-BET151 on BRD4 and activating histone marks in human tumor cells with an intact SHH signal pathway but SHH autonomy to avoid potentially confounding effects of SHH. Activated histone marks are present in the human first intron and TSS region along with BRD4 occupancy. BRD4 occupied the human GLI1 promoter and first intron and could be driven off by the inhibitor I-BET151 as shown in Fig. 5. As in mouse cells, I-BET151 significantly reduced GLI1 expression in human tumor cells (Fig. 2in Supplemental materials). However, the levels of H3K27ac and H3K4me3 occupancy were not significantly changed by I-BET151 treatment, suggesting that occupancy is SHH dependent but not BRD4 dependent.

### 2.3. Putative GBS in human and mouse GLI1 5′ introns

In addition to the public data our sequencing previously identified six highly conserved GLI putative binding sites (GBS, Fig. 1) with 8/9 nt matching the consensus sequence (GACCACCCA). The GBS sequences lined up with peaks of sequence conservation in this region (Fig. 1). Starting with the human sequence as the reference there was greater than 80% sequence identity between human, mouse, rat, dog and cow in the region of five of the six GBS (GBS 1–5) and greater than 70% sequence identity in the region of the sixth GBS between human, dog and cow. Conservation did not extend beyond mammals (data not shown). The structure of the mouse GLI1 gene differs in that there is a second untranslated exon [34] and the second intron shows four putative GBS with 8/9 nt matching the consensus sequence in the conserved regions. When the conservation plot compared mouse to human with the mouse sequence as the reference the GBS sequences line up with peaks of maximum conservation (Fig. 3 in Supplemental materials). Importantly the precise GBS sequences in the mouse were the same as those in the human including the mismatched nt. (Table 1 in Supplemental materials).

### 2.4. GLI1 and GLI2 can bind consensus sequences found in the intron

**Fig. 6.**
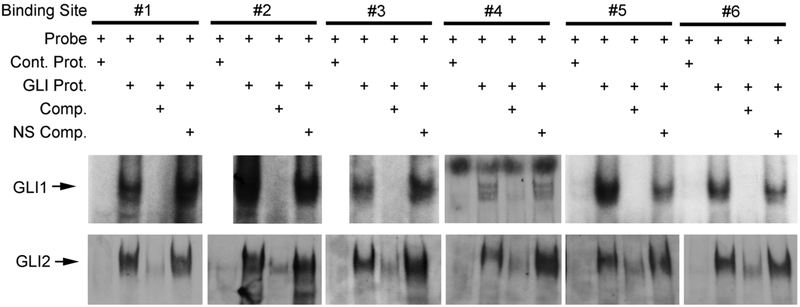
Gel shift assays demonstrate human GLI1 protein binds the putative GLI1 binding sites in the intronic region of human GLI1 gene. Labeled DNA probes were 29 mers including the 8/9 nt consensus sequence embedded, in the genomic sequence of the first intron. Electrophoretic mobility shift assays using purified human GLI1 or GLI2 protein demonstrate shifted bands (arrows) with specific oligonucleotide DNA probes (GBS1-6). The shifted bands are abrogated by > 100 fold molar excess of non-radiolabeled oligonucleotide. Control protein or a non-specific oligonucleotide DNA at > 100 fold molar excess did not affect the mobility shift, indicating the specificity of GLI-intron interaction. The lanes for GBS1, 2, and 3 with GLI1 are from the same gel utilizing the control probe shown in the first lane. They were separated in the figure to align with the appropriate lanes for GLI2.

We determined that both human GLI1 and GLI2 protein bind all six of the conserved GBS by gel shift analysis (Fig. 6). Purified human GLI1 protein (aa 211-1106) or purified GLI2 (aa. 84-355) partial proteins were incubated with the probes. Retarded bands were observed for all six conserved sites (arrow, Fig. 6).

### 2.5. Human GLI1 intron promotes transcriptional regulation of expression reporters by GLI1 and GLI2

**Fig. 7.**
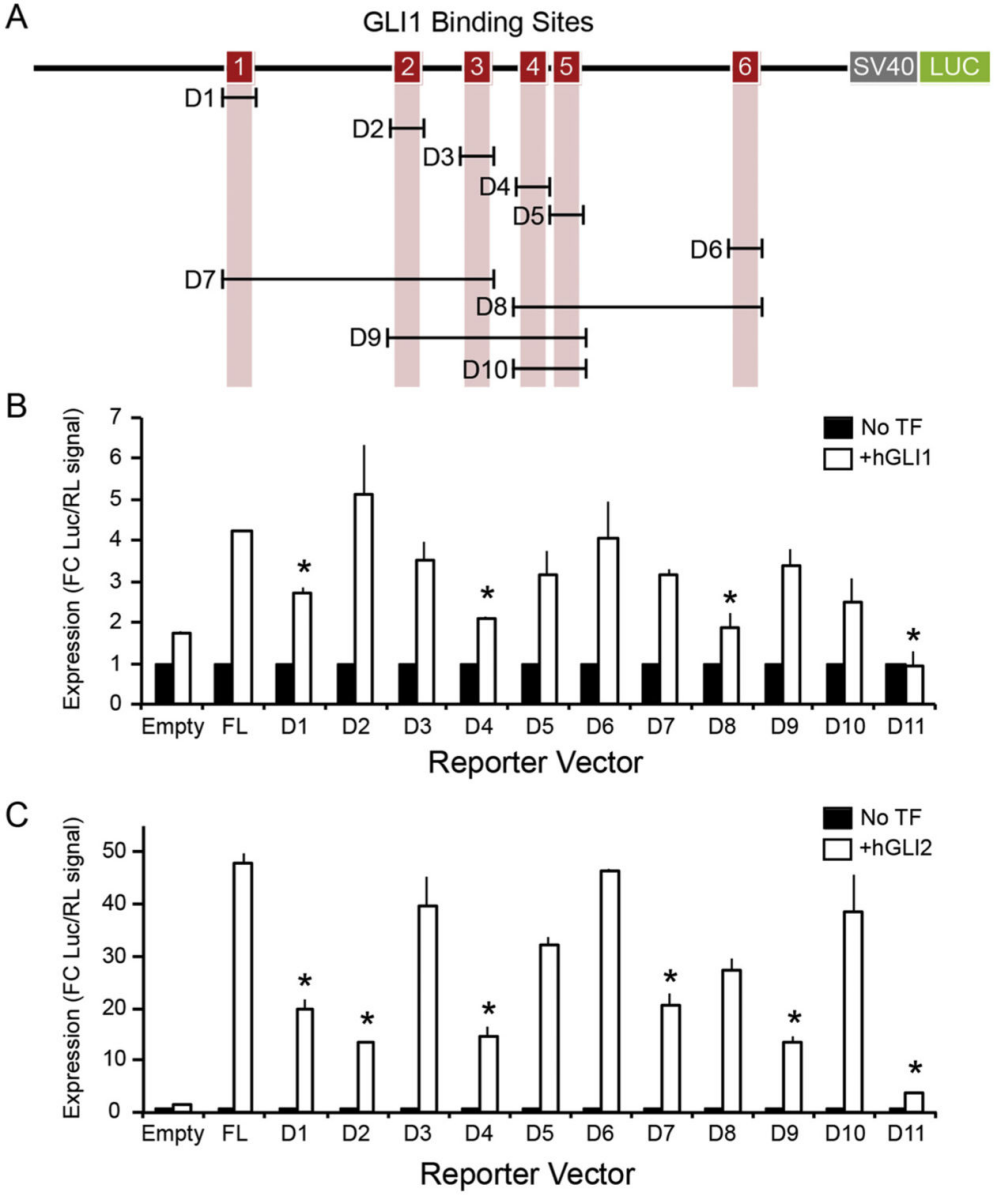
The hGLI1 intron acts as a transcriptional enhancer when stimulated by GLI transcription factors. **A.** Diagram showing 6 GLI binding sites (GBS, red boxes). A series of deletion mutants was generated by mutagenic inverse PCR to remove the bracketed sequences. D (for deletion) 1–10 remove the GBS individually and in combinations. D11 (not shown) removes the entire intron. **B and C.** Luciferase reporter assays demonstrate the ability of hGLI1 (**B**) and hGLI2 (**C**) to stimulate transcription with enhancer-like activity following transfection into HeLa cells. Luciferase signal is displayed as fold change upon addition of GLI transcription factor (open bars) over no transcription factor (closed bars). Error bars represent the S.E. of three independent experiments. Statistical significance (* = p < 0.05) between full length (FL) and deletion constructs is indicated. TF=transcription factor.

We then determined whether the first intron of the human GLI1 locus is competent to activate transcriptional activity through GLI1 or GLI2 binding to the GBS by cloning the full sequence of the intron into luciferase activity reporter vectors (Fig. 7A) utilizing HeLa human cells because they have no base line GLI1 expression and have been used extensively by us and others for plasmid based transduction allowing facile introduction and analysis of GLI expression and deletion constructs. In the presence of both human GLI1 and human GLI2 expression constructs, no activity above background was observed in either the basic vector or a vector designed to mimic promoter activity (pGL3:empty). However, in a vector designed to simulate enhancer activity (pGL3:in1A), both hGLI1 and hGLI2 stimulated transcription of the luciferase reporter gene (Fig. 7B,C). We deleted GBS individually to determine their contribution to GLI activity in the luciferase system. When hGLI1 was added we found that deletion of GBS1 (D1) or GBS4 (D4) (Fig. 7B) significantly reduced transcriptional activity. When hGLI2 was added GBS2 (D2) as well as GBS1 and GBS4 significantly reduced activity (Fig. 7C). We next deleted combinations of elements to expose groups of elements that may act in combination to promote transcriptional activity. When all GBS elements were deleted in a single construct (D11), almost all transcriptional activity was lost. When stimulated with hGLI1, we found that D8, which deleted GBS elements 4–6, but not D7, which deleted GBS elements 1–3, had significantly reduced activity indicating that GBS1 was less effective than GBS4. In contrast, when hGLI2 was added the reporter stimulation was significantly reduced in combination deletions D7 and D9 that removed GBS1, GBS2 and GBS4. However, D8 and D10 did not alter expression indicating that GBS4 was less effective. Thus we conclude that hGLI1 primarily acted through GBS1 and GBS4 while hGLI2 primarily acted through GBS1, GBS2 and GBS4.

### 2.6. Mass spectroscopy, proximity ligation assays and co-immunoprecipitation identify histone variant H2A.Z as a GLI1 binding partner

A GLI1-GFP construct was produced and activates transcription (data not shown) following transfection into HeLa cells. Proteins were isolated with GFP trap beads and separated on SDS PAGE gels and mass spectrometric analysis performed. In two independent isolations histone variant H2A.Z demonstrated high LFQ (label-free quantification) and high LFQ ratio between the GLI1-GFP and control plasmid pull down (Supplemental materials Fig. 4). The analysis also identified GLI1 (since that was the pull down antibody), SuFu (known to bind GLI1), and interestingly, SUV39H1, a histone methyltransferase considered to be a histone editor [37].

**Fig. 8.**
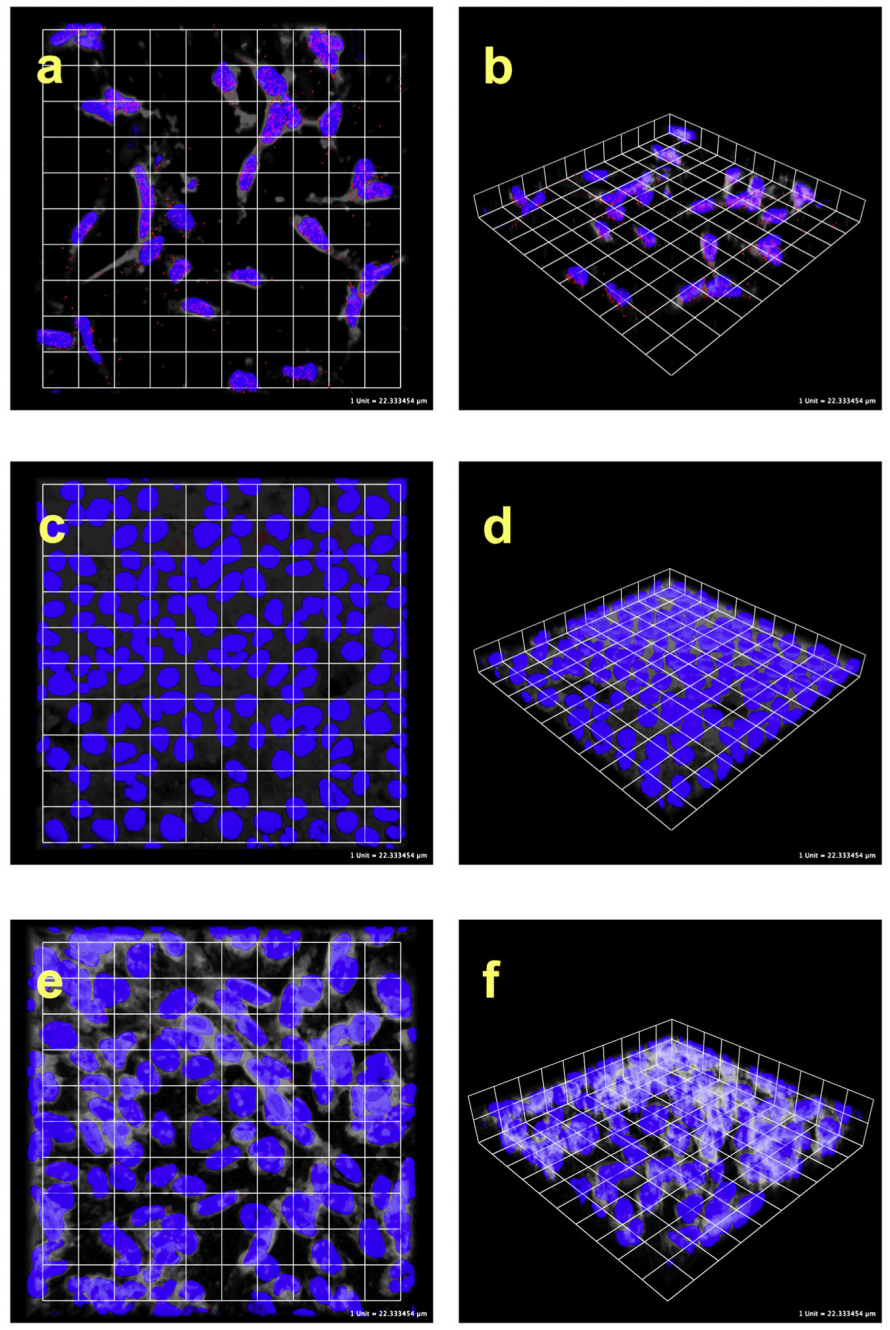
GLI1 binds H2A.Z. Confocal microscopy images of PLA and co-IP. **a** and **b** are Rh30 human rhabdomyosarcoma cells with high levels of GLI1, **c** and **d** are HeLa cells that lack GLI1, **e** and **f** are no primary antibody controls with Rh30 cells. Blue is DAPI nuclear stain, red is PLA signal, and white is processed brightfield data to allow visualization of approximate cell boundaries. PLA signal over the nuclei is present in **a** and **b**, but not in the controls **c-f**. Additional controls where only one of the primary antibodies is present likewise did not have PLA signal.

**Fig. 9.**
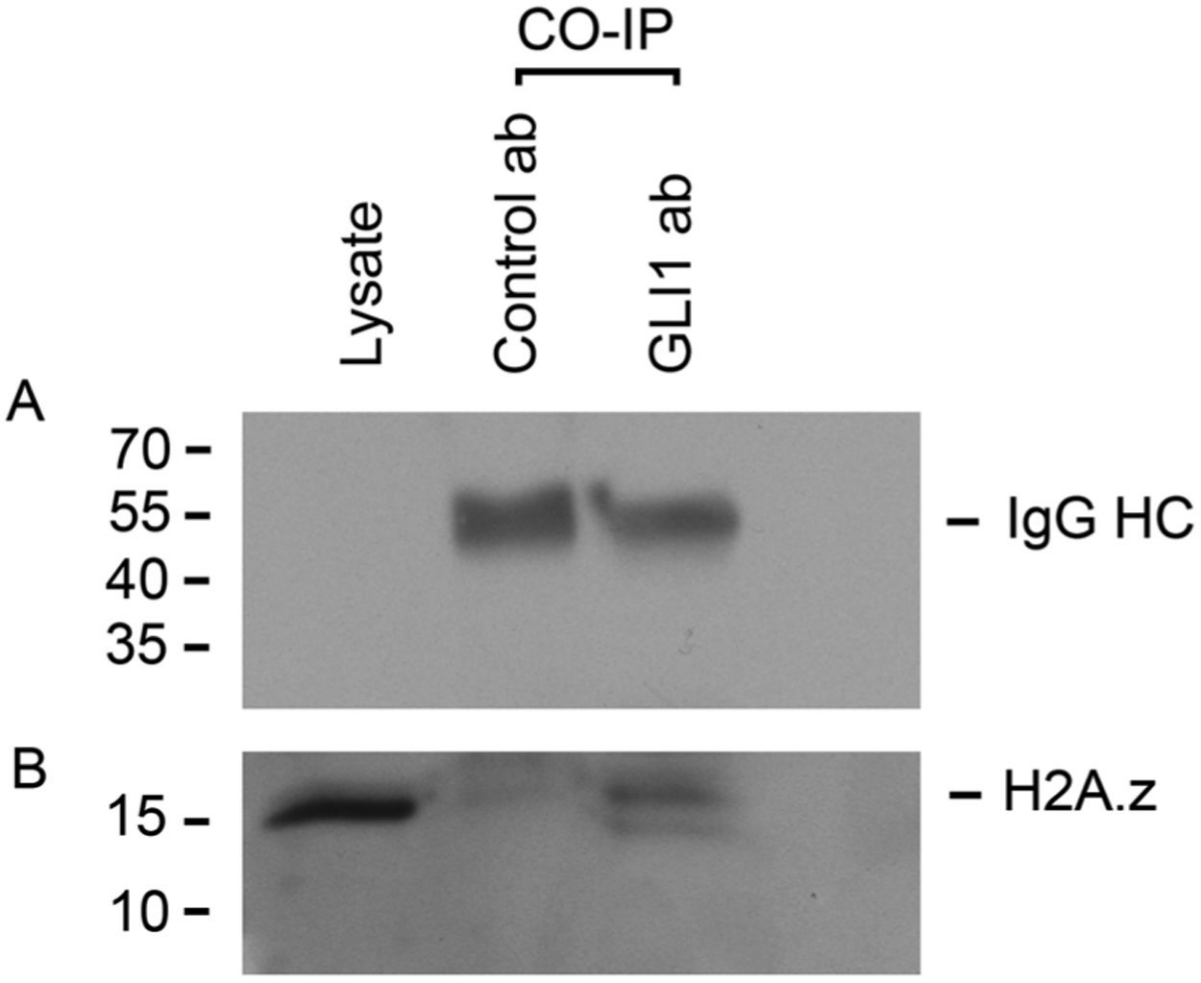
Co-immunoprecipitation (CO-IP) assay to detect the interaction between GLI1 and H2A.Z in Rh30 cells. GLI1 or control antibody (normal rabbit IgG) were used for CO-IP and H2A.Z was visualized following Western Blotting with anti-H2A.Z antibody. **A.** Heavy chain IgG bands show that the equivalent amount of each antibody was used. **B.** H2A.Z band is present in CO-IP lane with GLI1 antibody.

The interaction between GLI1 and histone H2A.Z was confirmed with proximity ligation assays (PLA; Fig. 8) utilizing Rh30 because of very high levels of GLI1 expression and protein levels, maximizing the opportunity to see the protein-protein interaction with a favorable signal to noise ratio. PLA is performed using primary antibodies from different species directed to the molecules of interest. The secondary antibodies are labeled with a DNA bar code and this is then recognized by circle forming DNA oligos. When the proteins are closer than 40 nm the oligos can be ligated and amplified by rolling circle amplification. The interaction is then visualized utilizing fluorescently labeled oligos that hybridize the circularized amplicon. This will result in hundreds fold amplification of signal and allows localization of the interaction. This allows the identification of protein-protein interactions without overexpression or use of exogenous labeled proteins or plasmids, and the method is highly sensitive and very specific. Cells were treated with antibodies to GLI1 and to H2A.Z. Using confocal microscopy PLA signal indicating binding of GLI1 and H2A.Z was observed in Rh30 cells (human rhabdomyosarcoma with amplified GLI1) illustrated in Fig. 8A and B. HeLa cells, which do not have detectable GLI1, did not display PLA signal (Fig. 8C and D). Co-immunoprecipitation confirmed the interaction between GLI1 and H2A.Z (Fig. 9). GLI1 antibody, but not normal rabbit IgG, pulled down H2A.Z identified with western blot analysis.

Public ChIP seq data showed multiple peaks of H2A.Z reads in the human GLI1 first intron in trophoblast, hES cells, mesenchymal stem cells derived from hES, neural progenitor cells derived from hES cells, HeLa, A549, HepG2 cells and a variety of others (Fig. 5, Supplemental materials). The H2A.Z read probability was maximal in the regions containing at least four of the six human GBS sequences in the GLI1 first intron.

## 3. Discussion

Here we show that the first intron of the human GLI1 gene is a regulatory region. Further, the histones present in the mouse and human GLI1 introns carry the active marks of H3K27ac and H3K4me3. Both GLI2 occupancy of the regulatory region and active epigenetic histone marks are SHH dependent. This SHH effect requires GLI2 and/ or GLI3 as it is absent in GLI2/GLI3 null cells. The human first intron contained six conserved GBS in regions of high sequence conservation that are capable of binding GLI1 and GLI2 in vitro. Deletion analysis demonstrates that binding to some but not all of the GBS significantly affects gene expression and that overall the elimination of all sites greatly reduces gene reporter expression. We show that SHH drives occupancy of GLI2, H3K27ac and H3K4me3 in this region and that the human intron can regulate GLI1 expression. The intron contains elements capable of activation of GLI1 expression explaining in part GLI1 auto-regulation.

While the epigenetic landscape is consistent with a nucleosome free region our work and the public data demonstrate the presence of histones within the intron and indeed our results show binding of GLI1 to H2A.Z, a developmentally relevant histone. The region is primed for remodeling and the histone reader protein BRD4 is present. H3K27ac and H3K4me3 occupancy is not significantly changed by removing BRD4 from the human intron and transcription start site (TSS) region with the small inhibitory molecule I-BET151 despite the fact that I-BET151 reduces GLI1 expression in both mouse [28] and human cells (Fig. 2 in Supplemental materials)

Concepts of transcriptional regulation through promoters and enhancers are rapidly evolving and the notion of a highly conserved cluster of GBS within the GLI genes themselves offers a potential mechanism for self-regulation by a positive feedback (feed forward) loop of the principal activating transcription factors in the pathway, GLI1 and GLI2. This region then is an ideal location to further study both function and regulation of GLI1. Interruption of the feedback loop could be exploited in novel cancer therapy. Additionally, the regulatory region has been shown in the public data to bind dozens of other transcription factors. Interestingly GBS6 can be seen to line up in ChIP seq with the largest number of transcription factors (Fig. 2 in Supplemental materials). Our data using cellular extracts rather than purified protein (not shown) reveals that GBS6 does not bind GLI1 or GLI2 in some cellular contexts although the purified proteins do bind that site. Elimination of the GBS6 site does not affect expression of the reporter gene in the presence of GLI1 or GLI2. It is possible given the other protein-protein interactions described here that the ability of that site to bind GLI1 or GLI2 is impeded by occupancy of other transcription factors.

Accumulating evidence suggests that transcriptional regulation occurs in a landscape of protein complexes designed to not only create a platform for transcriptional machinery but to facilitate the formation of elongating RNA. This has led to the concept of transcriptional factories [38]. The role of protein-protein interactions in the regions associated with transcriptional regulation may be to facilitate movement of the polymerase machinery through nucleosome organized DNA or alternatively the movement of DNA through the polymerase machinery. While the concept of nucleosome free regions allowing the RNAPol II complex to initiate may be overly simplistic, nucleosomes do present a barrier to RNA elongation [39]. The presence of histone reader proteins in the region of TSS may facilitate the polymerase activity through nucleosomes that remain in a transcriptionally active area or in the gene itself [40,41]. Bromodomain and extra terminal domain proteins bind acetylated histone tails though the bromodomain, a highly conserved 110 amino acid domain that forms alpha helices and breaks through proximal promoter pausing. BRD4 in particular may have histone chaperone activity that facilitates progression of RNAPII [42].

The presence of direct GLI1 and histone H2A.Z interaction is surprising. H2A is one of the four core histone proteins and is known to have several important variants. The H2A.Z variant is required for embryonic development and its knock out is embryonic lethal. Incorporation of H2A.Z into nucleosomes weakens the DNA winding and positioning of such nucleosomes around transcriptional start sites affects expression of the associated genes [43,44]. H2A.Z variant histones are important in gene activation and silencing and are highly conserved among species [45,46]. H2A.Z influences chromatin remodeling, occupies extended regions at developmental genes [47] and is an oncogene when over-expressed, possibly as a result of enhancing the expression of oncogenes like GLI1 [48-50]. Enrichment of H2A.Z is thought to help decrease pausing [51] leading to active transcription [52].

In humans, a significant cancer burden is associated with mis-regulation of the Hedgehog/Patched/GLI (HH/PTCH/GLI) pathway [1,53]. GLI1 gene targets sustain proliferation [10], inhibit apoptosis [10], promote angiogenesis [54] and promote tumor cell migration [55]. Wild-type p53 competes with GLI1 for the co-activator TAF9, inhibiting GLI’s oncogenic activity [56].

Transcription factors have proven to be difficult therapeutic targets because of specificity, selectivity and differential sensitivity [57]. Overcoming those concerns or generating new therapeutic approaches for GLI1 will require a better understanding of the biochemical mechanisms that regulate GLI1 gene expression and function. While the optimal GLI binding sequence is widespread in the genome it is important to realize that nt mismatches from the consensus are in many cases important to the functional regulation of gene targets [58]. The importance of the base pair differences from the consensus optimal sequence in the GBS described here is underscored by their conservation from human to mouse suggesting that these differences are more significant than just a relaxed binding domain. The cluster of GBS in the proximal intronic region of GLI1 is reminiscent of a similar structure in the Drosophila patched gene where it is thought to be part of a fine tuning response system and is evolutionarily conserved [59].

Mechanisms of control of GLI1 expression are important because GLI gene transcription is auto-regulated. Mouse GLI3 directly binds to the GLI1 promoter and induces GLI1 transcription in response to SHH.[60] Other experiments suggest that GLI1 is a direct target of GLI2 [61]. The results presented here imply that GLI1 can regulate the expression of GLI1 itself. Since non-canonical signaling may result in oncogenic expression of GLI1, inhibiting upstream molecules like SMO may not be useful for cancer therapy. Molecular inhibitors of GLI1 that directly affect its transcription by breaking the feed forward cycle may be required for meaningful therapy. A deeper understanding of the elements that regulate GLI1 expression will help achieve this goal.

## 4. Experimental procedures

### 4.1. Chromatin immunoprecipitation (ChIP)

10 million cells were cross-linked in 1% formaldehyde (Thermo scientific) for 10–15 min in room temperature and then blocked by 0.125 mM glycine (Ameresco) for 5 min in room temperature. Cell pellets were harvested and washed with pre-chilled PBS twice. Then 350 μl of lysis buffer was added to lyse cells. Cell lysates were then sonicated (Bioruptor™ UCD-200) to yield DNA fragments that have sizes between 300 and 800 base pairs. Thereafter, cell lysate were cleared after 10 min at 15,000 xg centrifugation. Supernatants were collected and diluted with ChIP dilution buffer by 10 fold. Diluted lysates were then precleared by protein A/G beads (Invitrogen) that had been blocked by BSA (Sigma) and salmon sperm DNA (Trevigen). Pre-cleared lysates were then aliquoted and incubated with 4 μg ChIP antibodies for 1 h. Then pre-blocked A/G beads were added for overnight incubation. On the second day, the beads were spun down and washed with low salt wash buffer, high salt wash buffer and lithium chloride buffer sequentially. Elution buffer was then added to elute protein-DNA complexes from the beads. Then the eluates were subjected to reverse crosslinking, RNA digestion (RNAse A: Sigma) and protein digestion (Proteinase K, Invitrogen) sequentially. A DNA purification kit (QIAGEN) was employed to purify DNA from previous steps. Purified DNAs were then utilized for q-PCR to quantify the abundance of associated proteins in specific regions of the gene locus.

### 4.2. Antibodies

BRD4 (Bethyl lab, Cat # A301-985A50), Rabbit IgG (Abcam, Cat # 46540), H3K27ac (Abcam, Cat # ab4729) and H3K4me3 (Abcam, Cat # ab1012), GLI2 (R&D, Cat # AF3526), Myc (Covance, Cat # MMS-150 P).

### 4.3. Cell lines

LIGHT2 (LT2) cells [62], *Gli2*^−/−^ *Gli3*^−/−^ MEFs [63], BL1648 cells [14] were used in these experiments.

### 4.4. Buffers

ChIP lysis buffer (1% SDS, 10mM EDTA, 50mM Tris, pH 8.0), ChIP dilution buffer (0.01% SDS, 1.2mM EDTA, 16.7mM Tris, 167mM NaCl, 1% Triton X-100), low salt wash buffer (0.1% SDS, 1% Triton X-100, 2mM EDTA, 150mM NaCl, 20 mM Tris, pH 8.0), high salt wash buffer (0.1% SDS, 1% Triton X-100, 2mM EDTA, 500mM NaCl, 20mM Tris, pH 8.0), Lithium chloride buffer (0.25M LiC1, 1% NP40, 1% deoxycholic acid, 1mM EDTA, 10mM Tris pH 8.0), elution buffer (1% SDS, 100mM NaHCO_3_).

### 4.5. Primers

Mouse GLI1 locus:

PS1Forward primer: GAGCAGACACCATGACCAAA Reverse primer: TTGGTTGGCCCAGGTAGTAGPS2Forward primer: TCCAGAATTGGAAGGCTCAC Reverse primer: GCCCAAGGATCTAGCAGTTGPS3Forward primer: AAGACCCCAAAGGCTCATCT Reverse primer: GTGGCAGCTCATCACAGAAAPS4Forward primer: ATTCCCAGCAACCACATGAT Reverse primer: GAGGGCATCAGATCCCATTAPS5Forward primer: TAAGTTCGCCAGTGCAATCA Reverse primer: AGTTGGGGTTTGGGAGAAAGPS6Forward primer: AGGAGATGCTCTGACGCCTA Reverse primer: AGTTCCCTCTACCACGCAGAPS7Forward primer: AGGAGATGCTCTGACGCCTA Reverse primer: TGCAGAGAAAAGAAGGCACAPS8Forward primer: AGCTGGGGAGACCTTGTTTT Reverse primer: GGCCTCTACGGAGTTTCCTTPS9Forward primer: ACCCAGGAATCCAAGGTGTC Reverse primer: TCCTGAAAGCAGGCAGTAGCPS10Forward primer: CGCTGAGAGAGGGAAGAATG Reverse primer: AAAGGTTTTCTGGGCTGGATPS11Forward primer: GATTTCCCCCAAAACCAAAC Reverse primer: GTGGAACACACGGAAGGTCT

Human GLI1 locus:

PS12Forward primer: ACTACAGCCAGGGAGTGTGG Reverse primer: TGTGTCCTCTGCAACCAGTCPS13Forward primer: CAATGTGGTCAAGACGGATG Reverse primer: TCCCATAGGGGTCAAGTGAGPS14Forward primer: GGGGAGGAGGAAGCAGATAG Reverse primer: CTGGGAAAAACCAGGGAACTPS15Forward primer: AACCCACTGACCTTCCACAC Reverse primer: TTAGATTTGCATTGCCATCGPS16Forward primer: CTAGGGAAAGGGGCTTCAGT Reverse primer: CACCCTTTGGATGGAACTTGPS17Forward primer: GGTAACCCCAGGTGTGTGTC Reverse primer: TCCCCTAAAGCACAAGCATCPS18Forward primer: CGGCTGCTATAACCAGCAAC Reverse primer: CTCCTCCTCTCAGCACATCCPS19Forward primer: ACAGCAGCACCTTCTTCCTC Reverse primer: GGTTCCTGAGGGGAGTCTTCPS20Forward primer: CTCTGCCTCTCTGGGACATC Reverse primer: GGTGCCTGTAATCCCAGCTA

### 4.6. Deletion mutations

#### 4.6.1. Luciferase transcriptional activity assay

Full-length human GLI1 intron (chr12:57,460,202-57,463,664; assembly hg38 from Dec. 2013) was cloned from HeLa cell lysate and ligated into pGL3 luciferase expression vectors using Gibson Assembly (NEB). Potential GLI binding sites (8/9 consensus bases) were identified using MacVector and deleted individually or in groups via inverse PCR. For luciferase assays, plasmids were cotransfected into HeLa cell culture using HilyMax (Dojindo) reagent. 30 h post transfection, cells were lysed and relative luciferase and renilla signal was detected using the Dual-Luciferase Reporter Assay system (Promega) with a Lumat luminometer (Berthold). After subtracting baseline noise and normalizing signal against renilla activity, statistical significance was determined by ANOVA (R).

Deletion primers:

TargethGLI1 intron1Forward Primer: GCCTGGGGTGAGACATTAGA Reverse Primer: CGCCTGTAATTCCAACGCTTD1Forward Primer: TCTGAATCCTCTTTCAGGT Reverse Primer: AGGAGGGGCCTCGATCCCCD2Forward Primer: AAGGCGGGACCGGGAGTAG Reverse Primer: ACTGGCTGCAGACGGCTCCD3Forward Primer: TGTAGCCCCATTTCCTTGG Reverse Primer: AGGCTAGTAAAAGGAAAATGD4Forward Primer: TCCGCACCTCGGTTGGAAAAG Reverse Primer: GACCTTGGAACTAATGTTGD5Forward Primer: CCGGCCCGCTCCCGGTGG Reverse Primer: TGGGATTTAGGGTGAGGGCD6Forward Primer: ACCCAGGCAAAGCTCCCAC Reverse Primer: TGTTTTGGACTAATTGTGCD7Forward Primer: TGTAGCCCCATTTCCTTGG Reverse Primer: AGGAGGGGCCTCGATCCCCD8Forward Primer: ACCCAGGCAAAGCTCCCAC Reverse Primer: GACCTTGGAACTAATGTTGD9Forward Primer: CCGGCCCGCTCCCGGTGG Reverse Primer: ACTGGCTGCAGACGGCTCCD10Forward Primer: CCGGCCCGCTCCCGGTGG Reverse Primer: GACCTTGGAACTAATGTTGD11Forward Primer: ACCCAGGCAAAGCTCCCAC Reverse Primer: AGGAGGGGCCTCGATCCCC

### 4.7. Electrophoretic mobility shift assays (EMSA)

EMSA was performed using DIG gel shift kit (Roche, Mannheim, Germany) or with ^32^P labeled probe and the GLI1 protein preparation was described previously [64]. Preparation of GLI2 Protein: 6xHisTag GLI2 protein was produced in *Escherichia coli* using the pETBlue-2 blunt cloning kit (Novagen, Madison, WI). The GLI2 aa 84–355 cDNA was prepared by PCR (sense primer:5′- GGAGCAGCTGGCTGACCTCAAG GAA-3′ and antisense 5′- CATCTCCACGCCACTGTCATTGTTG-3′) and cloned into the EcoRV site of the pETBlue-2 plasmid DNA (Novagen). For protein production, pETBlue-2 GLI2 construct was introduced into the Tuner (DE3)pLacI competent cells (Novagen) and GLI2 protein was induced with 1–2 mM isopropyl-1-thio-1-D-galactopyranoside (IPTG) for 3–4 h at 37°C. Bacteria were then harvested, sonicated, and cleared by centrifugation. The 6xHisTag GLI2 aa 84–355 in the clear lysate was purified using His-Spin Protein Miniprep kit with (Zymo Research, Irvine, CA) with modifications. EMSA was performed using DIG gel shift kit. 1 μl of GLI2 or control protein was used and the rest of the method is the same with GLI1 procedure.

5 μl of GLI1 protein (aa 211-1106) or control protein (pinpoint protein) was mixed with 2 μl of 5X binding buffer (Roche), H_2_O, and 0 or 1 μl (20 pmol) of unlabeled competitor oligonucleotides. The mixture was incubated at 4° C for 10 min. 1 μl (155 fmol) of double stranded digoxigenin-labeled probe was added and the mixture was incubated at 4°C for 20 min. Probes were designed using MacVector software (MacVector, Inc., Cary, NC). Probe sequences are shown, listing the sense sequence (5′→3′) followed by the antisense sequence (5′→3′) used to produce the double-stranded probe. We used the following probes (GBS sequences are in the Supplemental materials Table 1). GBS #1; sense 5′- CTCTGGCTCAGACCACCCTGCCTGCCCTT -3′ and antisense 5′- AAGGGCAGGCAGGGTGGTCTGAGCCAGAG -3′

GBS #2; sense 5′- GGCGGCGACTTGGGTGGGCCGAGGAGGCA -3′ and antisense 5′- TGCCTCCTCGGCCCACCCAAGTCGCCGCC -3′

GBS #3; sense 5′- AGGGGAGATATGGGTGGGCTGTGGAACGC -3′ and antisense 5′- GCGTTCCACAGCCCACCCATATCTCCCCT -3′

GBS #4; sense 5′- AGCATCCCGGGATCACCCACCGCGCCGGC -3′ and antisense 5′- GCCGGCGCGGTGGGTGATCCCGGGATGCT -3′

GBS #5; sense 5′- AAGGTCGAGTTGGGAGGTCTTGGATGCGG -3′ and antisense 5′- CCGCATCCAAGACCTCCCAACTCGACCTT -3′

GBS #6; sense 5′- TCTACACACAGACCACACAGGCAAAGCTC -3′ and antisense 5′- GAGCTTTGCCTGTGTGGTCTGTGTGTAGA -3′

non-specific competitor; sense 5′- TCTACACACAGACCACACAGGCAAAGCTC -3′ and antisense 5′- CGCAGACACACACCTGGGGTTACCTC -3′. The GLI1- DNA complexes were separated by 5% TBE gel electrophoresis, transferred onto Zeta-Probe GT membranes (Bio-Rad), and the shifted bands were visualized by anti-digoxigenin antibody and chemiluminescence reagent (Roche, Mannheim, Germany).

### 4.8. Mass spectrometric analysis

#### 4.8.1. GFP-GLI1 interaction screen

Adherent HeLa cells were transiently transfected with a plasmid encoding either GFP-GLI1 fusion protein or GFP alone under constitutive control of the CMV promoter. Expression of recombinant protein was confirmed visually via fluorescent microscopy and by western blotting. The fusion protein was shown to be functional with a luciferase GBS reporter (data not shown). Cultures were expanded to approximately 3.5 × 10^7^ cells and harvested via trypsinization. Cell pellets were then lysed with modified RIPA buffer (50mM Tris-HCl pH 7.5, 400mM NaCl, 1mM EDTA, 1% NP-40) and cleared by centrifugation. Lysates were then incubated with pre-cleared, equilibrated GFP-Trap beads (Chromotek) overnight at 4 °C. Next, beads were collected by centrifugation and washed with modified RIPA buffer (50mM Tris-HCl pH 7.5, 150mM NaCl, 0.1% NP-40, 1mM EDTA). Beads were then heated to 70 °C for 5 min in SDS loading buffer to elute proteins from beads. The supernatant was then collected by centrifugation and size-separated by SDS-PAGE. After gel fixation, lanes were divided by size and subjected to mass spec.

#### 4.8.2. Mass spectrometry

All mass spectrometric experiments were performed on a nanoscale UHPLC system connected to an Orbitrap Q-Exactive HF equipped with a nanoelectrospray source (all Thermo Fisher Scientific, Bremen, Germany). Each peptide fraction was auto-sampled and separated on a 15 cm analytical column (75 μm inner diameter) in-house packed with 1.9-μm C18 beads (Reprosil Pur-AQ, Dr. Maisch, Germany) using a 1 h gradient ranging from 5% to 40% acetonitrile in 0.5% formic acid at a flow rate of 250 nl/min. The effluent from the UHPLC was directly electrosprayed into the mass spectrometer. The Q Exactive HF mass spectrometer was operated in data-dependent acquisition mode and all samples were analyzed using the previously described ‘fast’ acquisition method [65]. All raw data analysis was performed with MaxQuant software suite [66] version 1.5.0.0 supported by the Andromeda search engine [67]. Data was searched against a concatenated target/decoy (forward and reversed) version [68] of the UniProt Human fasta database (downloaded from www.uniprot.org on 2014-01-23). Mass tolerance for searches was set to maximum 4.5 ppm for peptide masses and 20 ppm for HCD fragment ion masses. Data was searched with carba-midomethylation as a fixed modification. A maximum of three mis-cleavages was allowed while requiring strict trypsin specificity [69], and only peptides with a minimum sequence length of seven were considered for further data analysis. Peptide assignments were statistically evaluated in a Bayesian model on the basis of sequence length and Andromeda score. Only peptides and proteins with a false discovery rate (FDR) of less than 1% were accepted, estimated on the basis of the number of accepted reverse hits, and FDR values were finally estimated separately for modified and unmodified peptides [70]. Protein sequences of common contaminants such as human keratins and proteases used were added to the database. For LFQ quantification a minimum of two ratio-counts was required.

### 4.9. Proximity ligation assay (PLA)

PLA was performed as described by the manufacturer (Sigma-Aldrich, St. Louis, MO). Rh30 cells (gift from Dr. Peter Houghton, Greehey Children's Cancer Research Institute, San Antonio, TX), and HeLa cells (ATCC, Manassas, VA) were interrogated with anti-GLI1 antibody (R&D Systems, Minneapolis, MN) and/or anti-H2A.Z antibody (Cell Signaling, Danvers, MA) for PLA.

PLA labeled cells were imaged on a confocal microscope (Zeiss 510 META, Carl Zeiss, Jena, Germany). Z-stacks were acquired to allow for three-dimensional rendering in Volocity software (Perkin Elmer, Waltham, MA). DAPI fluorescence images were processed in the EBImage package or the R statistical programming environment to better define the nuclear boundary. Differential Interference Contrast data from brightfield images were processed in Photoshop (Adobe, San Jose, CA) using high pass filtering and median filtering to allow for rough visualization of cell boundaries. No processing was done to PLA fluorescence. All images were handled equivalently in Volocity, they were rendered in “3D Opacity” mode and channel opacity, density and black levels were all set to the same between images. Each square in the grid is approximately 22 μm on a side.

### 4.10. Co-immunoprecipitation assay

The following procedures were carried out at 4°C. Approximately 2 × 10 ^7^ Rh30 cells were resuspended and incubated in 1 ml of cell lysis buffer (Cell Signaling Technology) for 30 min with gentle rocking. The lysate was then homogenized by passing through a 26 G needle for several times and cleared by centrifugation for 10 min. at 10,000 rpm. The clear lysate was incubated with 20 μl of Anti-GLI1 antibody (Rockland) or control antibody (Cell Signaling Technology) for 30 min and then was incubated with 20 μl of protein A/G-magnetic beads (Thermo Scientific) for 30 min with gentle rocking. GLI1-H2A.Z Immune complex was collected by using a magnetic rack for 1 min, washed with lysis buffer, and then eluted with low pH IgG elution buffer (Thermo Scientific). The eluate was then neutralized with an equal volume of 1 M Tris.Cl (pH 7.6). All the following procedures were carried out at room temperature. The GLI1-H2A.Z eluate was separated on a SDS-PAGE gel, transferred onto a nitrocellulose membrane and incubated in PBST buffer with 5% milk for 30 min. The membrane was washed with PBST buffer, incubated with polyclonal rabbit H2A.Z antibody (Active Motif) (1:4000 dilution) in PBST buffer with 5% milk for 1 h. The membranes were washed with PBST buffer (1X PBS, 0.3% Tween-20) and incubated with secondary antibody conjugated with HRP (Donkey anti Rabbit IgG-HRP, Santa Cruz Biotech) for 1 h (1:8000 dilution in PBST with 5% milk). The membrane was then washed 3 times with PBST buffer. The H2A.Z protein was visualized using SuperSignal West Pico chemiluminescence kit (Thermo Scientific).

## Supplementary Material

01

02

03

04

05

06
