# Supplementary figures and images for: Regulation of GLI1 by cis DNA elements and epigenetic marks
Source: DNA Repair (Amst). Author manuscript; Available in PMC 2019 Jul 1. (PMC6570425; doi:10.1016/j.dnarep.2019.04.011)

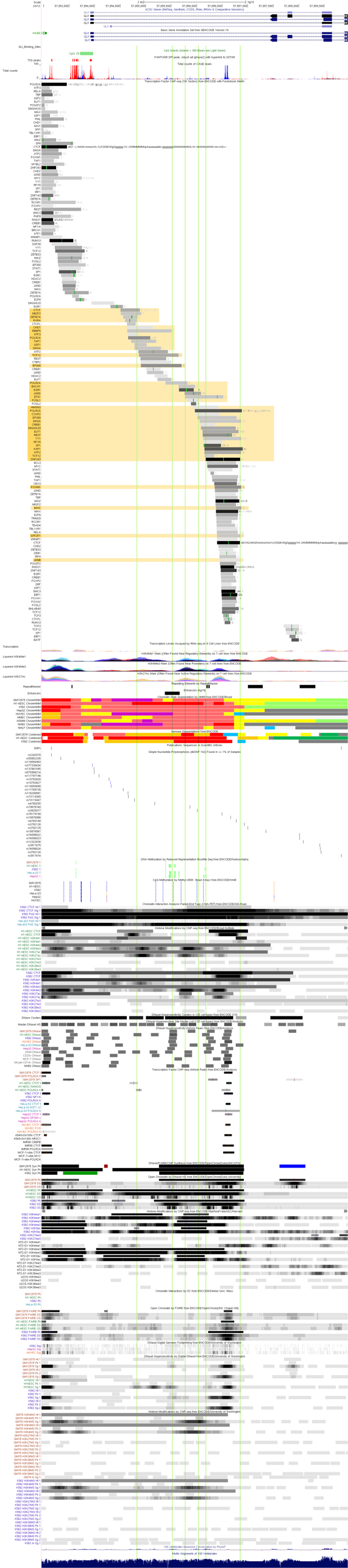

Supplement: 01 [file NIHMS1529566-supplement-01.pdf]

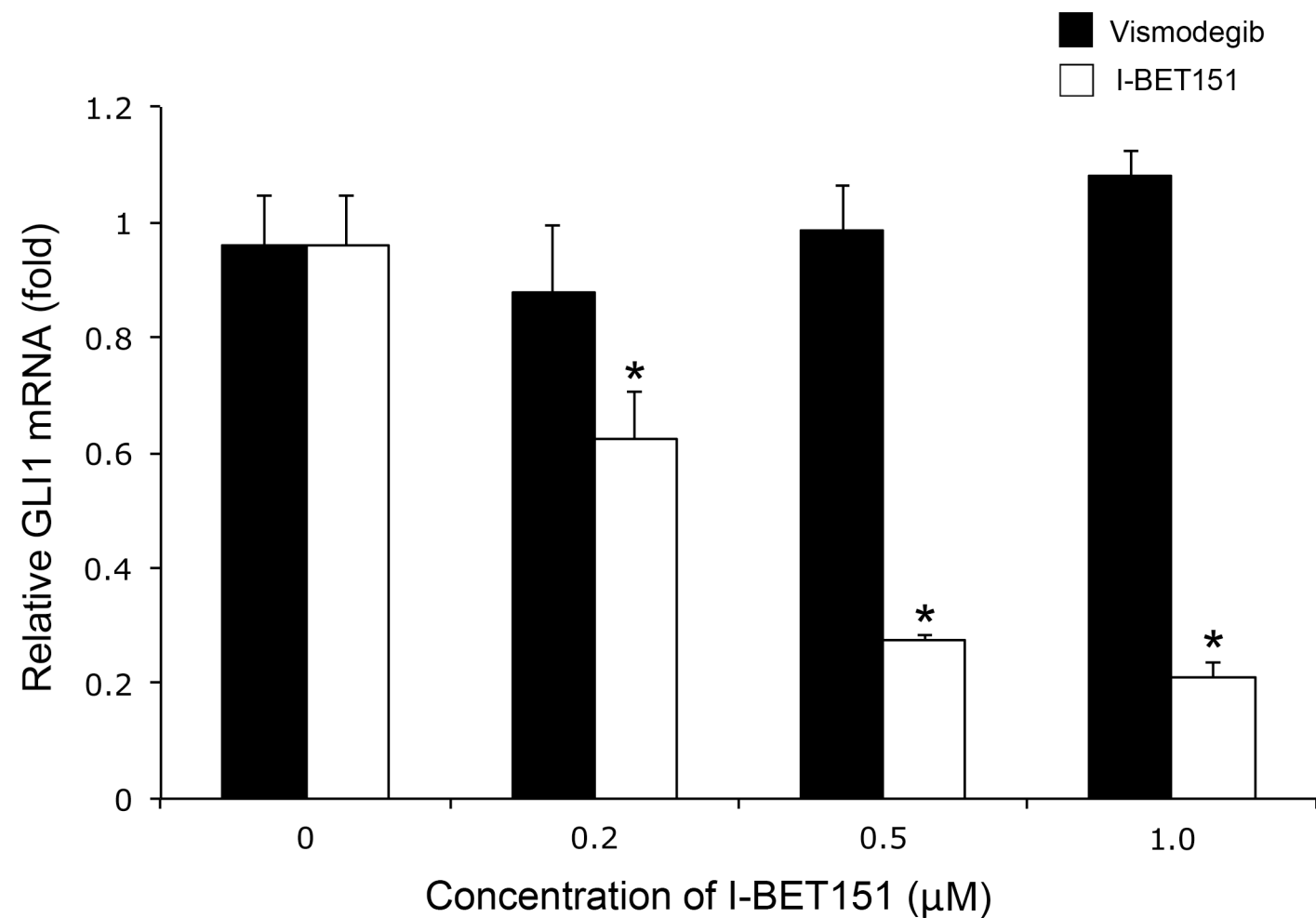

Supplement: 02 [file NIHMS1529566-supplement-02.pdf]

Mouse Dec. 2011 chr10:127338200-127341700

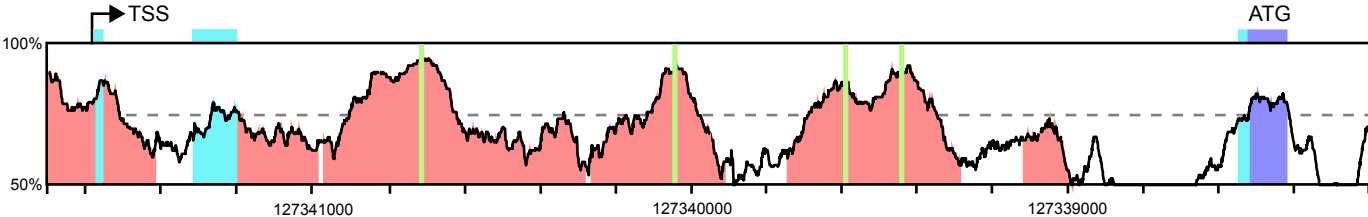

Supplement: 03 [file NIHMS1529566-supplement-03.pdf]

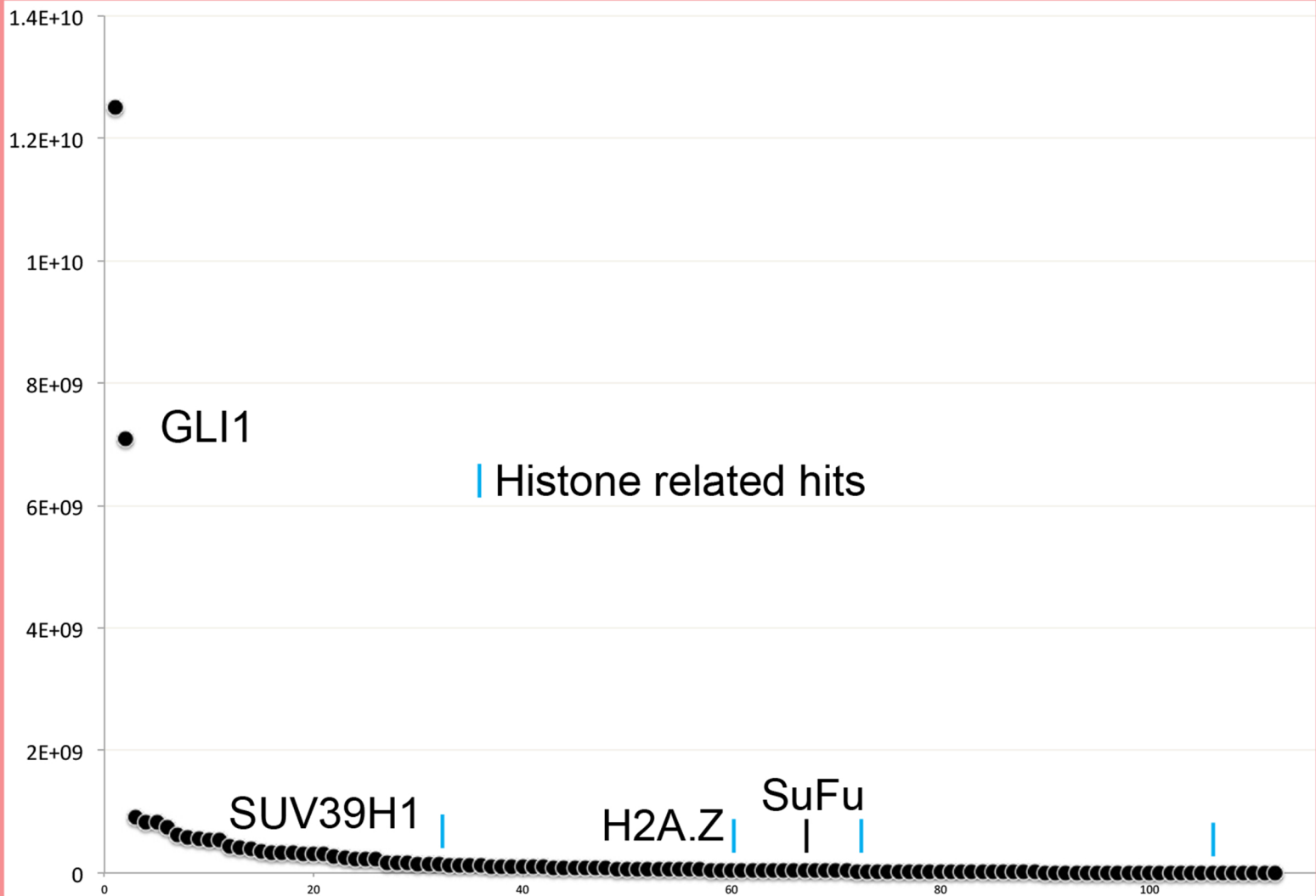

Supplement: 04 [file NIHMS1529566-supplement-04.pdf]
