# Supplementary material for: Regulation of GLI1 by cis DNA elements and epigenetic marks
Source: DNA Repair (Amst). Author manuscript; Available in PMC 2019 Jul 1. (PMC6570425; doi:10.1016/j.dnarep.2019.04.011)
Supplement: 06 [file NIHMS1529566-supplement-06.docx]

Supplemental Data:

Supplemental Table 1

HUMAN GBS in GLI1 FIRST INTRON^1^

| Site | nt Position relative to TSS | Sequence (lower case represents mismatch from consensus) |
| --- | --- | --- |
| GBS#1 | 919 | +GACCACCCt |
| GBS#2 | 1614 | -GcCCACCCA |
| GBS#3 | 1868 | -GcCCACCCA |
| GBS#4 | 2126 | -GACCtCCCA |
| GBS#5 | 2269 | +GAtCACCCA |
| GBS#6 | 2948 | +GACCACaCA |

MOUSE GBS in GLI1 SECOND INTRON^2,3^

| Site | nt Position relative to TSS | Sequence (lower case represents mismatch from consensus) |
| --- | --- | --- |
| GBS#1 | 867 | +GACCACCCt (identical to Human GBS #1) |
| GBS#2 | 1533 | -GcCCACCCA (identical to Human GBS #2) |
| GBS#3 | 1998 | -GACCtCCCA (identical to Human GBS #4) |
| GBS#4 | 2139 | +GAtCACCCA (identical to Human GBS #5) |

^1^ Search on Chromosome 12 locus NC_000012.12

^2^ Search on Chromosome 10 locus NC_000076.6

^3^The mouse GLI1 sequence includes an additional exon relative to the human sequence with a very short first intron, making the mouse second intron equivalent to the human first intron (34).

Supplemental Figure 1. Genome browser data with alignment to the conserved GBS. Track descriptions are taken from the UCSC browser data GRC37/hg19 (71). This is not the most recent assembly but the track data are native to this assembly.

GLI_Binding_Sites

Sites of GLI1 motif binding with at 8/9 nucleotide matching *(mapping in the 1^st^ intron only).* Six red lines through all tracks. (see Supplemental Table 1)

CpG Islands

Light green boxes mark identified CpG islands, which mark transcription start sites.

FANTOM5 DPI peaks

Public data from the RIKEN group for mapping transcriptional starts using CAGE *(cap analysis gene expressions)* reads. Red peaks match locations of active transcription, blue peaks match to reads on the opposite strand and together they should frame the nucleosome free region.

<http://fantom.gsc.riken.jp/5/>

Transcription Factor ChIP-seq

Covers 161 unique regulatory factors *(generic and sequence-specific factors)*, spanning 91 human cell types, some under various treatment conditions and represents peak calls *(regions of enrichment)* generated by the ENCODE Analysis Working Group *(AWG)* using the uniform processing pipeline developed for the ENCODE Integrative Analysis effort and published in a set of coordinated papers in September 2012. The darker the box, the higher the mapped reads for that transcription factor. Within a cluster, a green highlight indicates the highest scoring site of a Factorbook-identified canonical motif for the corresponding factor. Factors that overlap a GLI1 binding site are highlighted in yellow. Factors whose description mentions a zinc finger have a red asterisk.

<http://www.factorbook.org/human/>

Transcription

The data comprising these tracks were generated from hundreds of experiments on multiple cell lines conducted by labs participating in the ENCODE project. Each of the cell lines in a track is associated with a particular color. Light, saturated colors are used to produce the best transparent overlay.

Layered H3K4me1

Shows histone marks associated with regulatory elements, overlaid to show peaks for 7 cell lines *(GM12878, H1-hESC, HSMM, HUVEC, K562, NHEK, NHLF).*

Layered H3K4me3

Shows histone marks associated with promoters, overlaid to show peaks for 7 cell lines *(GM12878, H1-hESC, HSMM, HUVEC, K562, NHEK, NHLF).*

Layered H3K27ac

This track shows the levels of enrichment of the H3K27ac histone mark across the genome as determined by a ChIP-seq assay. The H3K27ac histone mark is the acetylation of lysine 27 of the H3 histone protein, and it is thought to enhance transcription possibly by blocking the spread of the repressive histone mark H3K27me3. *(Shown for 7 cell lines).*

Chromatin State Segmentation by HMM

Displays chromatin state segmentation of the human genome into fifteen states grouped to predict functional elements. This track displays a chromatin state segmentation for each of nine human cell types *(GM12878, H1-hESC, HepG2, HUVEC, HMEC, HSMM, K562, NHEK, NHLF)*. A common set of states across the cell types were learned by computationally integrating ChIP-seq data for nine factors plus input *(CTCF, H3K4me1, H3K4me2, H3K4me3, H3K27ac, H3K9ac, H3K36me3, H4K20me1, H3K27me3, and Input)* using a Hidden Markov Model *(HMM)*, a computer learning algorithm. In total, fifteen states were used to segment the genome, and these states were then grouped and colored to highlight predicted functional elements.

- Active Promoter
- Weak Promoter
- Inactive/poised Promoter
- Strong enhancer
- Strong enhancer
- Weak/poised enhancer
- Weak/poised enhancer
- Insulator
- Transcriptional transition
- Transcriptional elongation
- Weak transcribed
- Polycomb-repressed
- Heterochromatin; low signal
- Repetitive/Copy Number Variation
- Repetitive/Copy Number Variation

Single Nucleotide Polymorphisms

Known SNPs mapped with vertical lines with RefSNP identifiers for NCBI’s dbSNP build 142 and shows a subset of the single nucleotide polymorphisms and small insertions and deletions *(indels)*. Only SNPs that have a minor allele frequency of at least 1% and are mapped to a single location in the reference genome assembly are included in this subset. The selection of SNPs with a minor allele frequency of 1% or greater is an attempt to identify variants that appear to be reasonably common in the general population. Taken as a set, common variants should be less likely to be associated with severe genetic diseases due to the effects of natural selection, following the view that deleterious variants are not likely to become common in the population.

DNA Methylation

This track was produced as part of the ENCODE project. The track reports the percentage of DNA molecules that exhibit cytosine methylation at specific CpG dinucleotides. In general, DNA methylation within a gene's promoter is associated with gene silencing and DNA methylation within the exons and introns of a gene is associated with gene expression. Proper regulation of DNA methylation is essential during development and aberrant DNA methylation is a hallmark of cancer. DNA methylation status was assayed at more than 500,000 CpG dinucleotides in the genome using Reduced Representation Bisulfite Sequencing (RRBS). Genomic DNA was digested with the methyl-insensitive restriction enzyme MspI and then small genomic DNA fragments were purified by gel electrophoresis and used to construct an Illumina sequencing library. The library fragments were treated with sodium bisulfite and amplified by PCR to convert every unmethylated cytosine to a thymidine while leaving methylated cytosines intact. The sequenced fragments were aligned to a customized reference genome sequence. For each assayed CpG, the number of sequencing reads covering that CpG and the percentage of those reads that were methylated were reported.

Red – 100% of molecules sequenced are methylated

Yellow – 50%

Green – 0%

CpG Methylation

This track is produced as part of the ENCODE project. The track displays the methylation status of specific CpG dinucleotides in the given cell types as identified by the [Illumina Infinium Human Methylation 450 Bead Array platform](http://www.illumina.com/products/methylation_450_beadchip_kits.ilmn). In general, methylation of CpG sites within a promoter causes silencing of the gene associated with that promoter. The Infinium Human Methylation 450 platform uses bisulfite treated genomic DNA to assay the methylation status of more than 450,000 CpG sites covering all designatable RefSeq genes, including promoter, 5' and 3' regions, without bias against those lacking CpG islands. Additionally, the assay includes CpG islands and shores, CpG sites outside of CpG islands, non-CpG methylated sites identified in human stem cells, differentially methylated sites identified in tumor versus normal *(multiple forms of cancer)* and across several tissue types, CpG islands outside of coding regions, miRNA promoter regions, and disease-associated regions identified through GWAS.

orange = methylated (score >= 600)

purple = partially methylated (200 < score < 600)

bright blue = unmethylated (0 < score <= 200)

black = NA (score = 0)

Chromatin Interaction Analysis Paired-End Tags (ChIA-PET)

This track shows the locations of protein factor mediated chromatin interactions determined by ChIA-PET data in five different human cancer cell lines *[K562 (chronic myeloid leukemia), HCT116 (colorectal cancer), HeLa-S3 (cervical cancer), MCF-7 (breast cancer), and NB4 (promyelocytic)]*. The protein factors displayed in the track include estrogen receptor alpha, RNA polII, and CTCF. Chromatin interaction analysis with paired-end tag sequencing *(ChIA-PET)* is a global de novo high-throughput method for characterizing the 3-dimensional structure of chromatin in the nucleus. A chromatin interaction is defined as the association of two regions of the genome that are far apart in terms of genomic distance, but are spatially proximate to each other in the 3-dimensional cellular nucleus.

Histone Modification by ChIP-seq

**University of Washington:** This track shows genome-wide maps of histone modifications associated with active promoters *(H3K4me3)*, repressed regions *(H3K27me3)*, and active transcription *(H3K36me3)* in 57 cell types, as identified by ChIP-seq.

**ENCODE/Stanford/Yale/USC/Harvard:** adds 9 new experiments for the MCF-7, HCT-116 and PANC-1 cell lines.

**Broad Institute:** Adds 83 new experiments including 6 new cell lines and 25 new antibodies.

DNaseI Hypersensitivity / Open Chromatin

**Uniform Peaks:** This track displays the uniform set of open chromatin elements in 125 ENCODE cell types on a per-cell type basis.

**FAIRE *(Formaldelhyde-Assisted Isolation of Regulatory Elements)*:** This is a method to isolate and identify nucleosome-depleted regions of the genome.

**DNaseI Hypersensitivity Clusters:** This track displays clusters of Uniform DNaseI Hypersensitive sites across the cell types assayed based on DNase-seq data.

**DNaseI Digital Genomic Footprinting:** This track contains deep sequencing DNase data that can be used to identify sites where regulatory factors bind to the genome *(footprints)*. Footprinting is a technique used to define the DNA sequences that interact with and bind DNA-binding proteins, such as transcription factors, zinc-finger proteins, hormone-receptor complexes, and other chromatin-modulating factors like CTCF. The technique depends upon the strength and tight nature of protein-DNA interactions. This track contains a total of 22 DGF experiments, covering 20 mouse cell types and tissues.

**DNaseI Hypersensitivity by Digital DNaseI:** This track shows DNaseI sensitivity measured genome-wide in different cell lines using the Digital DNaseI methodology.

Cell Lines:

A549 Adenocarcinomic human alveolar basal epithelial cells

CD20+ CD20 positive B-cells

GM12878 Blood lymphoblastoid cells

GM78

H1-hESC Human embryonic stem cells (ICM)

HCT-116 Human epithelial colorectal carcinoma

HeLa Immortal cervical carcinoma cell line

HepG2 Liver carcinoma (endoderm lineage)

HMEC Human dermal microvascular endothelium

HSMM Human skeletal muscle and myoblasts

HUVEC Human umbilical vein endothelial cells (mesoderm lineage)

IMR90 Fetal lung fibroblasts

K562 Immortal chronic myelogenous leukemia cell line

MCF-7 Mammary gland adenocarcinoma

Mcyte-CD14+ CD14 positive cells from leukapheresis production

NB4 Acute promylocytic leukemia with t(15;17) (q22;q11-12)

NHEK Normal human epidermal keratinocytes

NHLF Normal human lung fibroblasts

NT2-D1 Human testis; derived from metastatic site: lung (NTERA-2 cl.D1)

PANC-1 Human pancreas duct epithelium

U-2 OS Human Osteosarcoma (epithelial)

Supplemental Figure 1. I-BET151 significantly reduces GLI1 expression in human BL1648 (ATCC, Manassas, VA) human lymphoma cells. BL1648 cells were treated with the SMO inhibitor Vismodegib (LC lab, Woburn, MA; 0- 0.2 µM in DMSO, to inhibit the canonical HH pathway as a negative control) or with the bromodomain inhibitor I-BET151 (Tocris, Minneapolis, MN; 0-1 µM in DMSO) for 24 hours. Relative GLI1 expression was measured by qPCR, threshhold cycle number of GLI1/GAPDH with solvent only is 1 fold. Experiments were performed in triplicate. * represents statistical significance (Student t-test, p<0.05).

Supplemental Figure 2. Mouse conservation plot relative to human sequence. The transcription start site (TSS) and translation start (ATG) are labeled. % Sequence identity in 100 nt windows is represented on the Y-axis. 5’->3’ is left to right. The green bars represent the positions of the mouse GBS (8/9 nt match to consensus, Supplemental Table 1).

Supplemental Figure 3. LFQ (y-axis) for proteins identified by IP mass spec. GLI1 is identified as the pull down was done with GLI1 antibody. SuFu is a known GLI1 binder. H2A.Z is discussed. SUV39H1 is “histone related”, and is a histone methyltransferase and as such is considered a histone editor (37).

Supplemental Figure 4. Public human H2A.Z ChIP-seq data from the UCSC Genome Browser. Green lines represent the positions of the six human 8/9 consensus GBS sequences. Cell lines are displayed in the left column.
