# Supplementary material for: Regulation of GLI1 by cis DNA elements and epigenetic marks
Source: DNA Repair (Amst). Author manuscript; Available in PMC 2019 Jul 1. (PMC6570425; doi:10.1016/j.dnarep.2019.04.011)
Supplement: 05 [file NIHMS1529566-supplement-05.pdf]

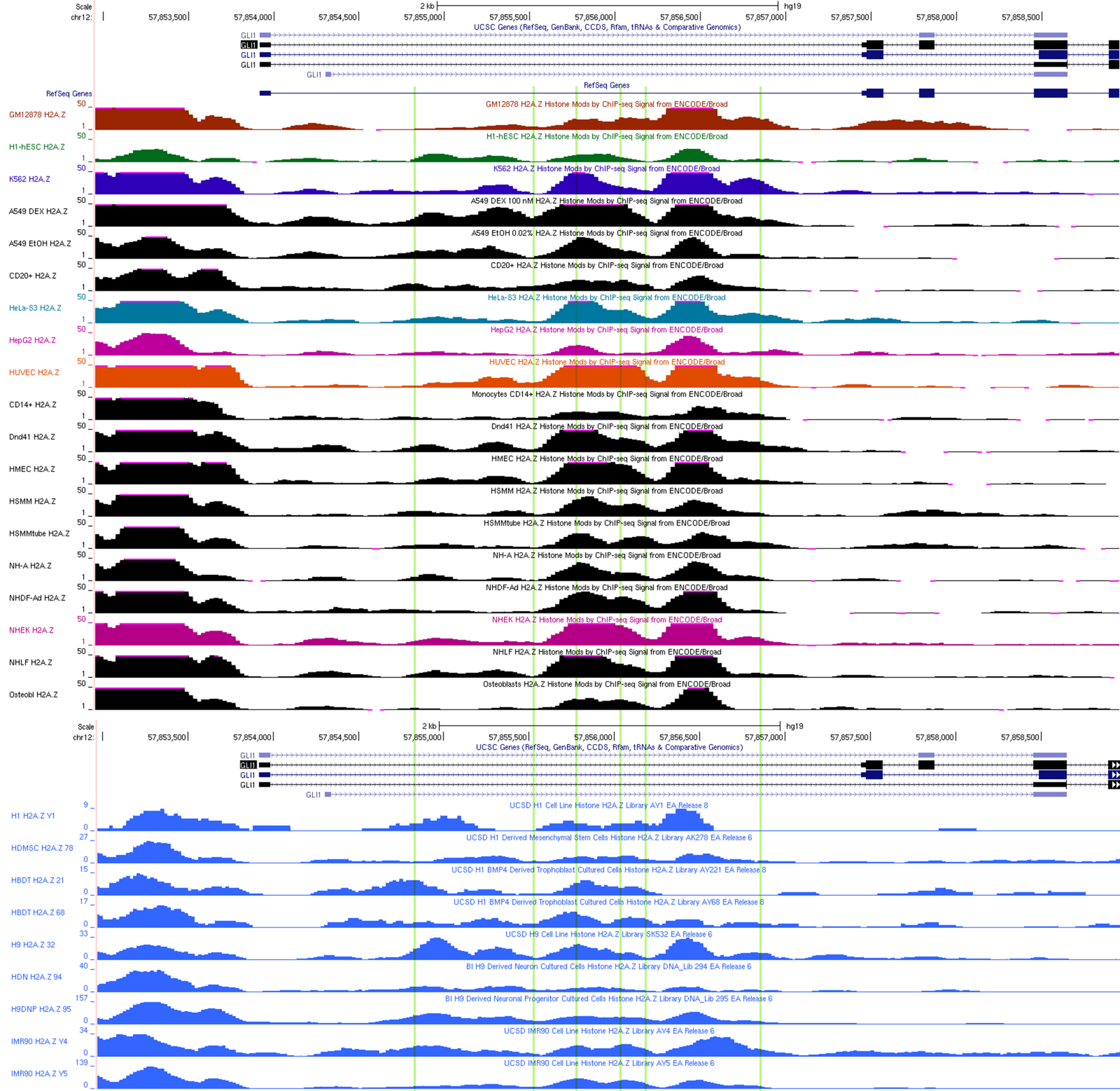
Scale
chr12:
2 kb
hg19
57,853,500
57,854,000
57,854,500
57,855,000
57,855,500
57,856,000
57,856,500
57,857,000
57,857,500
57,858,000
57,858,500
UCSC Genes (RefSeq, GenBank, CCDS, Rfam, tRNAs & Comparative Genomics)
GLI1
GLI1
GLI1
GLI1
GLI1
RefSeq Genes
RefSeq Genes
GM12878 H2A.Z
GM12878 H2A.Z Histone Mods by ChIP-seq Signal from ENCODE/Broad
H1-hESC H2A.Z
H1-hESC H2A.Z Histone Mods by ChIP-seq Signal from ENCODE/Broad
K562 H2A.Z
K562 H2A.Z Histone Mods by ChIP-seq Signal from ENCODE/Broad
A549 DEX H2A.Z
A549 DEX 100 nM H2A.Z Histone Mods by ChIP-seq Signal from ENCODE/Broad
A549 EtOH H2A.Z
A549 EtOH 0.02% H2A.Z Histone Mods by ChIP-seq Signal from ENCODE/Broad
CD20+ H2A.Z
CD20+ H2A.Z Histone Mods by ChIP-seq Signal from ENCODE/Broad
HeLa-S3 H2A.Z
HeLa-S3 H2A.Z Histone Mods by ChIP-seq Signal from ENCODE/Broad
HepG2 H2A.Z
HepG2 H2A.Z Histone Mods by ChIP-seq Signal from ENCODE/Broad
HUVEC H2A.Z
HUVEC H2A.Z Histone Mods by ChIP-seq Signal from ENCODE/Broad
CD14+ H2A.Z
Monocytes CD14+ H2A.Z Histone Mods by ChIP-seq Signal from ENCODE/Broad
Dnd41 H2A.Z
Dnd41 H2A.Z Histone Mods by ChIP-seq Signal from ENCODE/Broad
HMEC H2A.Z
HMEC H2A.Z Histone Mods by ChIP-seq Signal from ENCODE/Broad
HSMM H2A.Z
HSMM H2A.Z Histone Mods by ChIP-seq Signal from ENCODE/Broad
HSMMtube H2A.Z
HSMMtube H2A.Z Histone Mods by ChIP-seq Signal from ENCODE/Broad
NH-A H2A.Z
NH-A H2A.Z Histone Mods by ChIP-seq Signal from ENCODE/Broad
NHDF-Ad H2A.Z
NHDF-Ad H2A.Z Histone Mods by ChIP-seq Signal from ENCODE/Broad
NHEK H2A.Z
NHEK H2A.Z Histone Mods by ChIP-seq Signal from ENCODE/Broad
NHLF H2A.Z
NHLF H2A.Z Histone Mods by ChIP-seq Signal from ENCODE/Broad
Osteobl H2A.Z
Osteoblasts H2A.Z Histone Mods by ChIP-seq Signal from ENCODE/Broad
H1 H2A.Z Y1
UCSD H1 Cell Line Histone H2A.Z Library AY1 EA Release 8
HDMSC H2A.Z 78
UCSD H1 Derived Mesenchymal Stem Cells Histone H2A.Z Library AK278 EA Release 6
HBDT H2A.Z 21
UCSD H1 BMP4 Derived Trophoblast Cultured Cells Histone H2A.Z Library AY221 EA Release 8
HBDT H2A.Z 68
UCSD H1 BMP4 Derived Trophoblast Cultured Cells Histone H2A.Z Library AY68 EA Release 8
H9 H2A.Z 32
UCSD H9 Cell Line Histone H2A.Z Library SK532 EA Release 6
HDN H2A.Z 94
BI H9 Derived Neuron Cultured Cells Histone H2A.Z Library DNA_Lib 294 EA Release 6
H9DNP H2A.Z 95
BI H9 Derived Neuronal Progenitor Cultured Cells Histone H2A.Z Library DNA_Lib 295 EA Release 6
IMR90 H2A.Z Y4
UCSD IMR90 Cell Line Histone H2A.Z Library AY4 EA Release 6
IMR90 H2A.Z Y5
UCSD IMR90 Cell Line Histone H2A.Z Library AY5 EA Release 6
